# Supplementary material for: Building synthetic chromosomes from natural DNA
Source: Nat Commun. 2023 Dec 20;14:8337. doi: 10.1038/s41467-023-44112-2 (PMC10733283; doi:10.1038/s41467-023-44112-2)

Supplementary Information for

Building synthetic chromosomes from natural DNA

Alessandro L.V. Coradini, Christopher Ne Ville, Zachary Krieger, Joshua Roemer, Cara Hull, Shawn Yang, Daniel T. Lusk, and Ian M. Ehrenreich

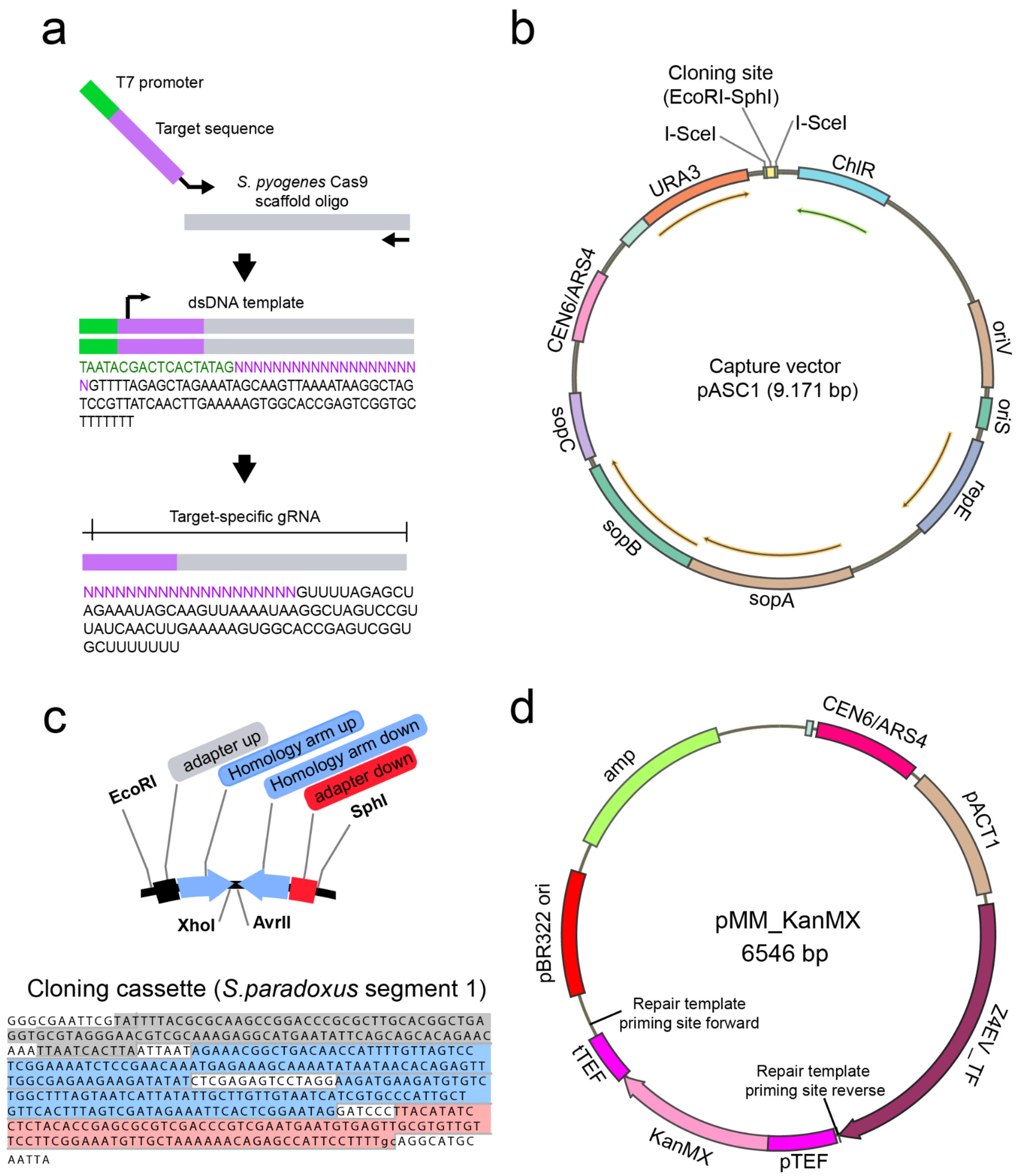

Supplementary Figure 1: Reagents used for cloning in CReATiNG. a. DNA templates for producing gRNAs by in vitro transcription are generated by PCR. An oligonucleotide containing the tracrRNA is amplified using a tailed forward primer containing a 20 nt target sequence and a T7 promoter. The PCR reaction generates a dsDNA template that is transcribed in vitro into gRNAs by T7 RNA polymerase. **b.** Map of the BAC/YAC vector pASC1. The vector contains a cloning site flanked by I-SceI where a cloning cassette is inserted using restriction digestion and ligation. **c.** Example of a cloning cassette design for capture of *S.paradoxus* segment 1. The cassette contains segment-specific homology arms that are flanked by adapters that program how cloned segments

will recombine during chromosome assembly. Once the cloning cassette is added to pASC1, the vector is linearized by restriction digestion, exposing the homology arms. **d.** Map of the modified pRS316 vector, which has *URA3* replaced by *KanMX* (pMM_*KanMX*). PCR of *KanMX* with tailed primers is used to make repair templates for cloning reactions. Primers bind to the sites labeled as repair template priming sites.

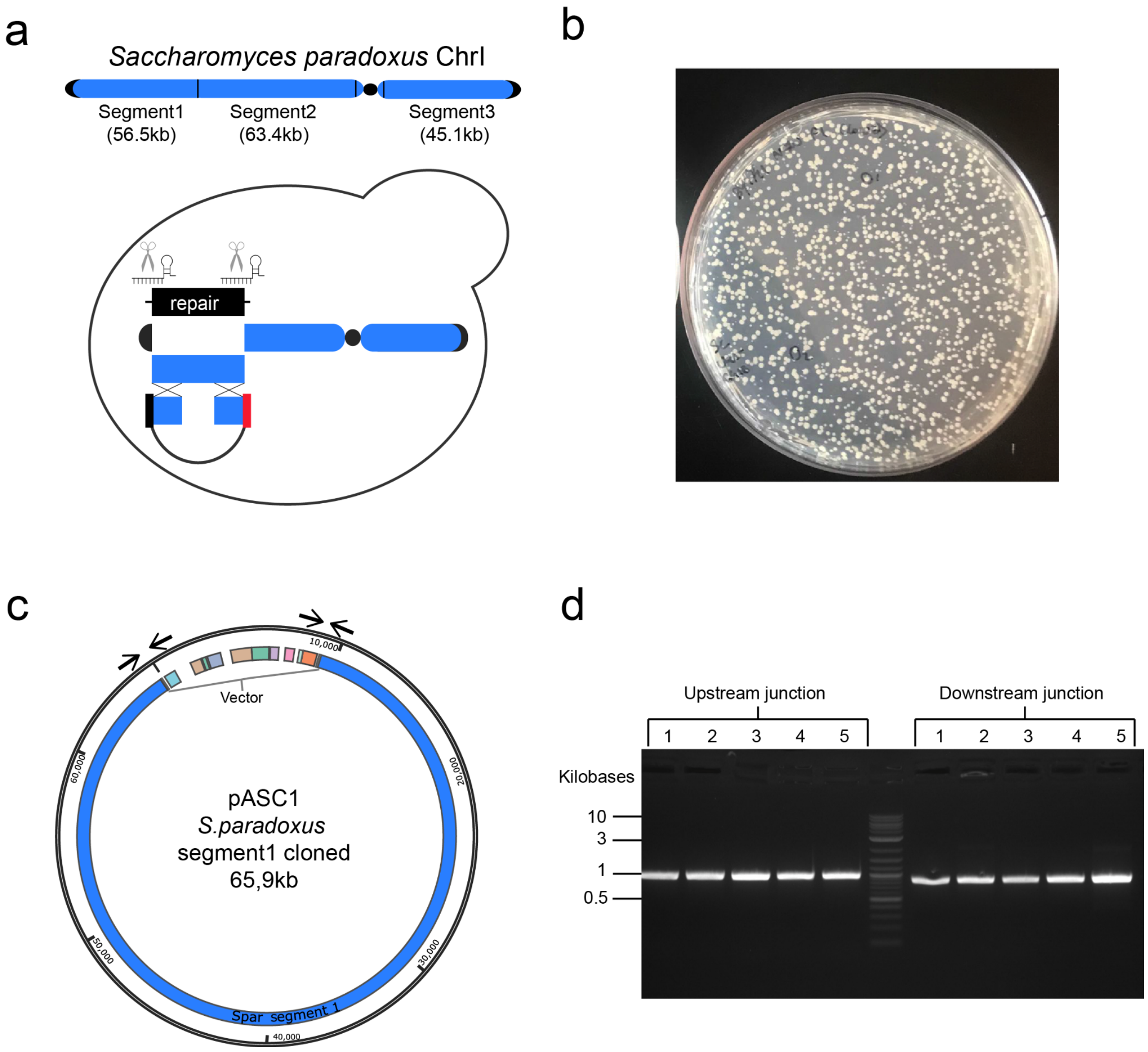

Supplementary Figure 2: The cloning step of CReATiNG has high efficiency. a. Map of the strategy for cloning the core of *Saccharomyces paradoxus* ChrI as three segments. **b.** Transformed donor cells are selected on SC plates lacking uracil and containing G418. **c.** Map of the pASC1 cloning vector containing segment 1 from *Saccharomyces paradoxus* ChrI. The black arrows indicate primer sites for PCR junction checks. **d.** Cloning efficiency was accessed using junction PCRs. Electrophoresis in a 1% agarose gel shows the expected DNA bands for both junctions in 100% (5 of 5) colonies checked for cloning of segment 1.

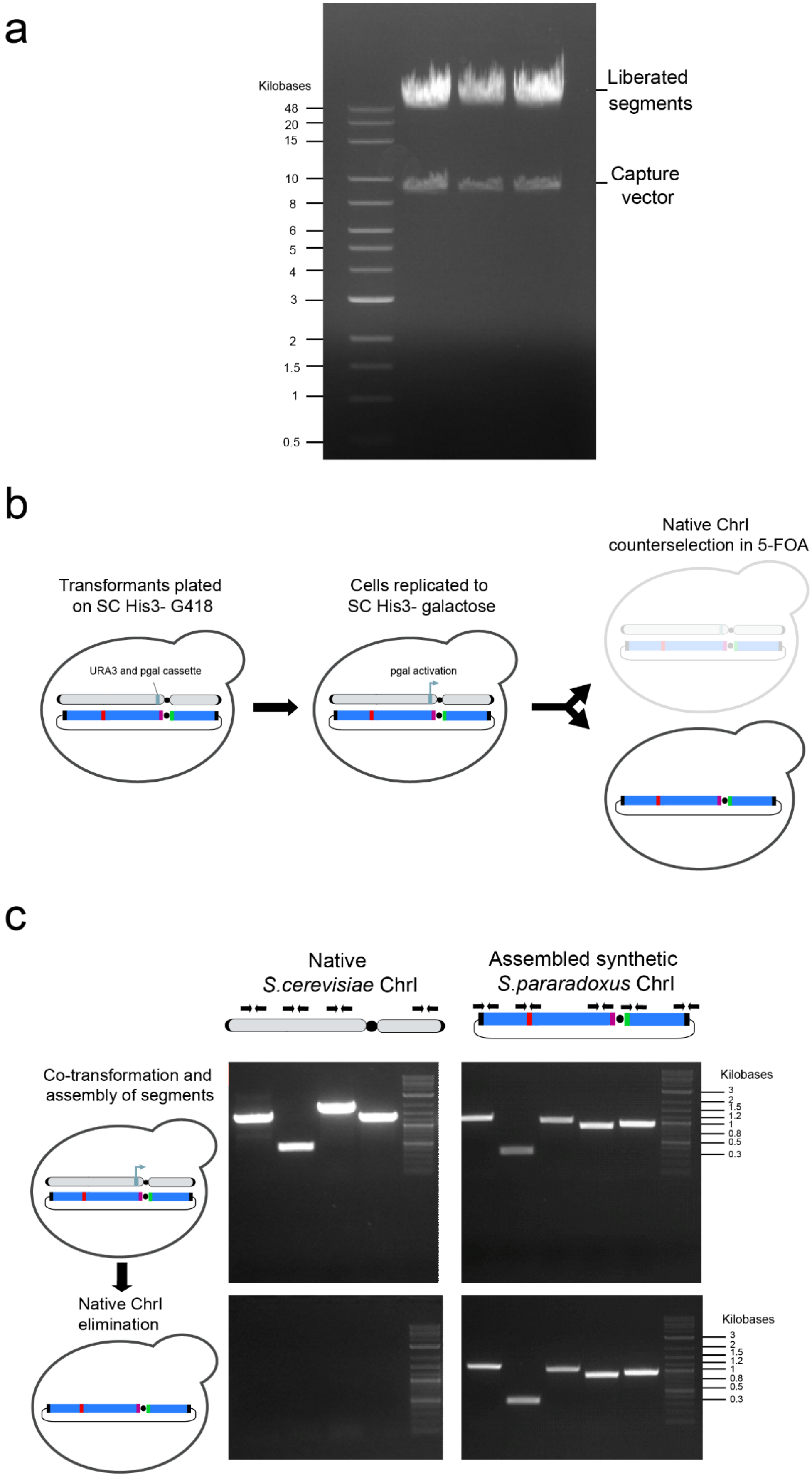
a
Kilobases
48
20
15
10
8
6
5
4
3
2
1.5
1
0.5
Liberated segments
Capture vector
b
Transformants plated on SC His3- G418
URA3 and pgal cassette
Cells replicated to SC His3- galactose
pgal activation
Native ChrI counterselection in 5-FOA
c
Native S.cerevisiae ChrI
Assembled synthetic S.pararadoxus ChrI
Co-transformation and assembly of segments
Native ChrI elimination
Kilobases
3
2
1.5
1.2
1
0.8
0.5
0.3

Supplementary Figure 3: Assembly of a synthetic *S.paradoxus* ChrI in BY. **a.** The three segments of *S.paradoxus* ChrI were liberated from the cloning vector by I-SceI digestion and separated from the vector in an 0.5% agarose gel. **b.** The three segments, the assembly vector (pASC2), and a centromere cassette were co-transformed into and assembled in BY. The native BY ChrI was marked for elimination prior to assembly. **c.** Correct assembly of *S.paradoxus* ChrI and elimination of the native ChrI were initially confirmed by diagnostic PCRs targeting both chromosomes. The gel pictures on the left show junction PCR results for native BY ChrI before (upper) and after (bottom) native ChrI elimination. The gel pictures on the right show junction PCR results for the *S.paradoxus* ChrI before (upper) and after (bottom) native ChrI elimination.

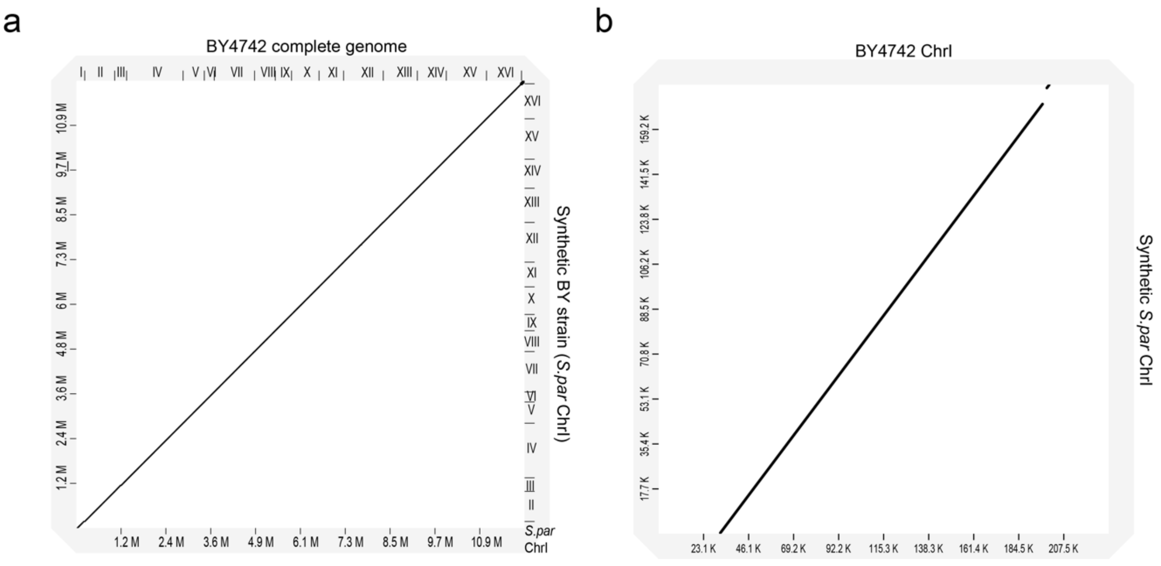

Supplementary Figure 4: Dot plots comparing the genomes of BY containing *S.paradoxus* ChrI and the progenitor BY. a. CReATiNG did not introduce any structural changes genome-wide. **b.** Comparison of *S.paradoxus* ChrI to BY ChrI reveals that synteny was maintained during chromosome assembly. The gap in the upper right is caused by a natural length difference between BY and *S.paradoxus* in segment 3.

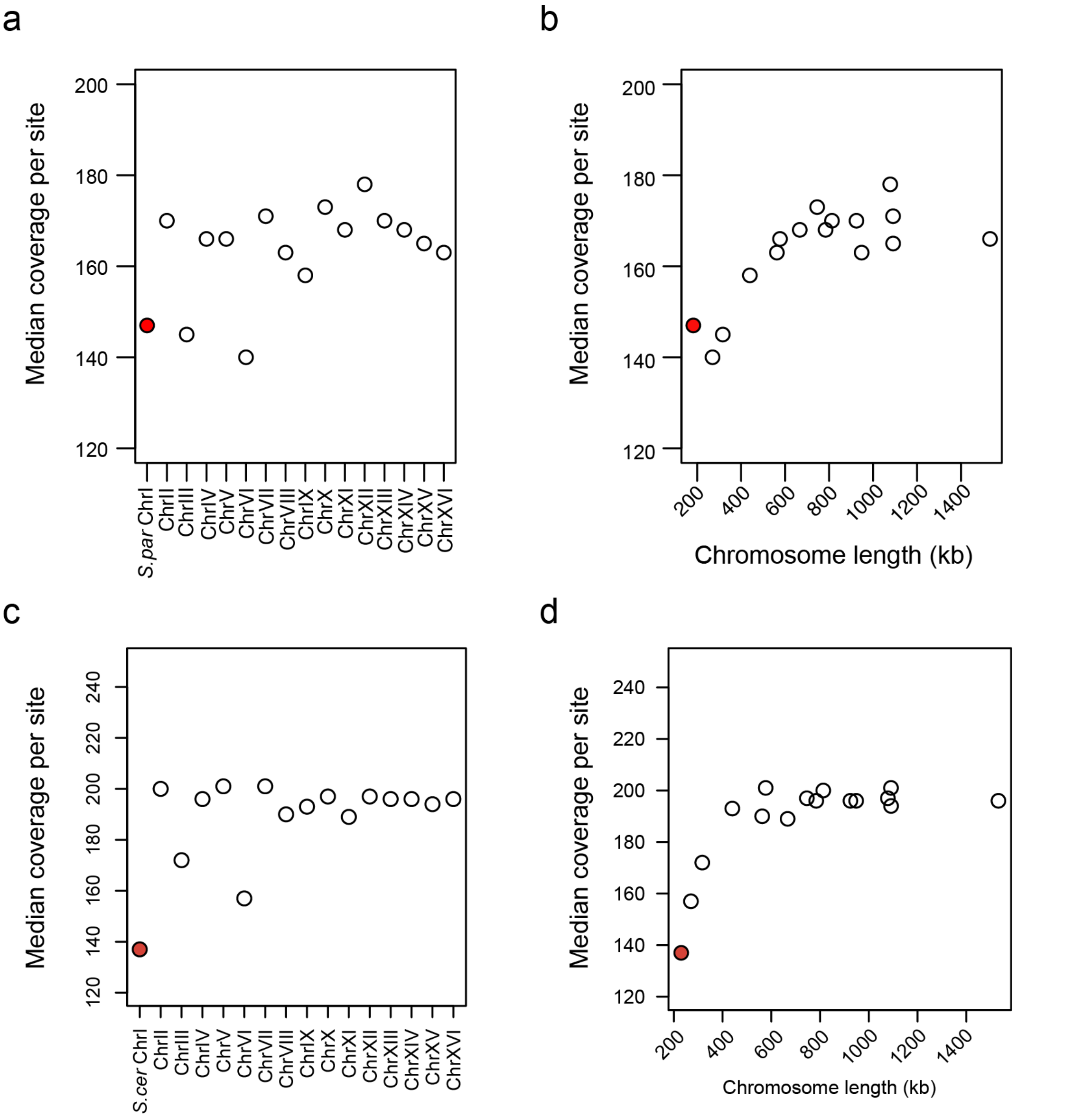

Supplementary Figure 5: Genome-wide per site coverage analysis for all chromosomes in the euploid strain containing *S.paradoxus* ChrI and its progenitor. a. Plot showing median per site coverage for each chromosome in the IEY394 strain, which is a euploid strain possessing *S.paradoxus* ChrI in an otherwise *S.cerevisiae* genome. **b.** Plot showing the relationship between median coverage per site and chromosome length (kb). In **a** and **b**, coverage values corresponding to the synthetic *S.paradoxus* ChrI are highlighted in red. **c.** Plot showing median per site coverage for each chromosome in the BY4742 parental strain prior to chromosome substitution. **d.** Plot showing the relationship between median coverage per site and chromosome length (kb). In **c** and **d**, coverage values corresponding to the native *S.cerevisiae* ChrI are highlighted in red. The data used in **a-d** was generated using Oxford Nanopore Technologies long read

sequencing of high molecular weight genomic DNA extractions. The DNA extractions were biased towards larger chromosomes, producing differences in coverage between smaller and larger chromosomes. The key insight from these plots is that *S.paradoxus* ChrI shows similar coverage to the other native, small chromosomes in the cell and to the native ChrI in a progenitor cell, suggesting it is present at one copy per cell in IEY394.

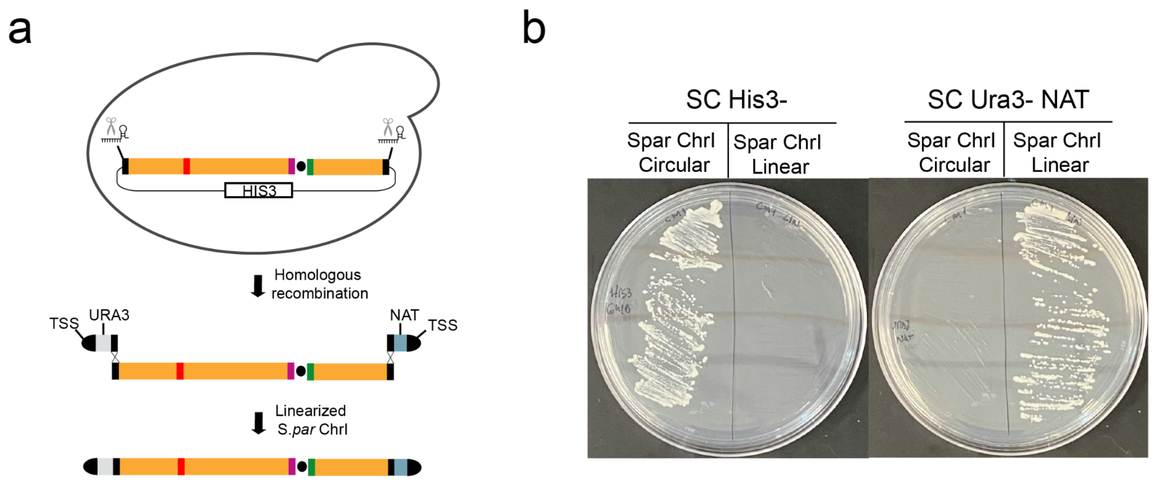

Supplementary Figure 6: *S.paradoxus* ChrI linearization using CRISPR/Cas9. a. We assembled chromosomes as circular molecules. To linearize the synthetic *S.paradoxus* ChrI, we targeted the junctions between the chromosome and the vector with CRISPR/Cas9. We also provided repair templates containing synthetic telomere seed sequences (TSS) and selectable markers, *URA3* and *NatMX* for the left and right arms, respectively. **b.** Plate images showing cells in which the circular *S.paradoxus* ChrI was converted to its linear form. Cells with the linear ChrI become histidine auxotrophs, but are uracil prototrophs with resistance to nourseothricin.

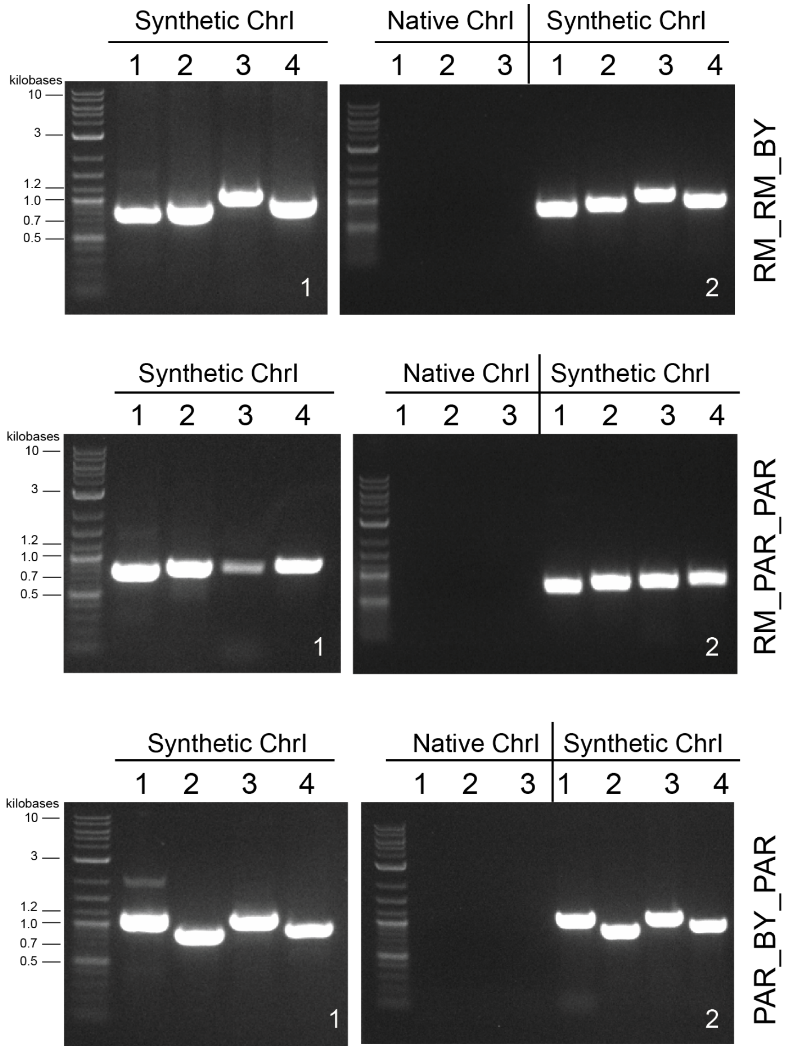

Supplementary Figure 7: Assembly of recombinant versions of ChrI using segments from multiple donors. The gel pictures on the left (1) show junction PCRs for three distinct assemblies prior to elimination of native ChrI, with donors of each segment noted. The gel pictures to the right (2) show junction PCRs for the same three assemblies after elimination of native ChrI. These gels contain 1% agarose. All native chromosome checks in the aneuploids were positive, as expected (not shown).

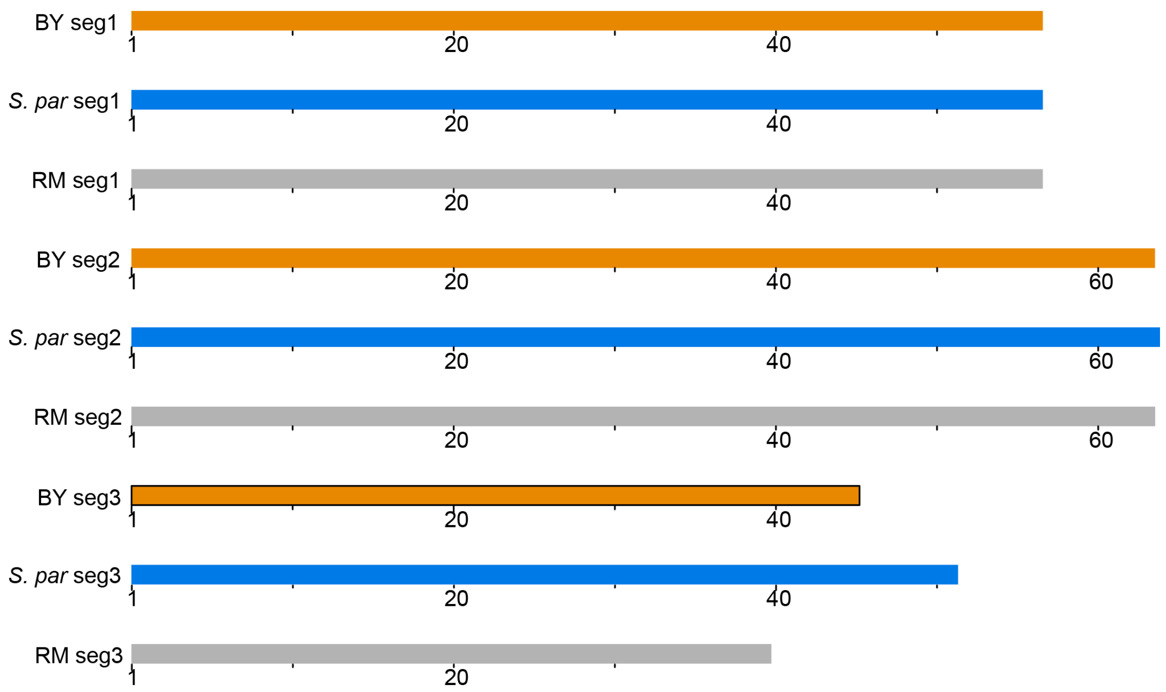

Supplementary Figure 8: Cloned segments from the core of ChrI. BY (orange), RM (gray) and *S.paradoxus* (blue) chromosome segments 1 and 2 show similar sizes across strains and species. However, segment 3 is more variable in size.

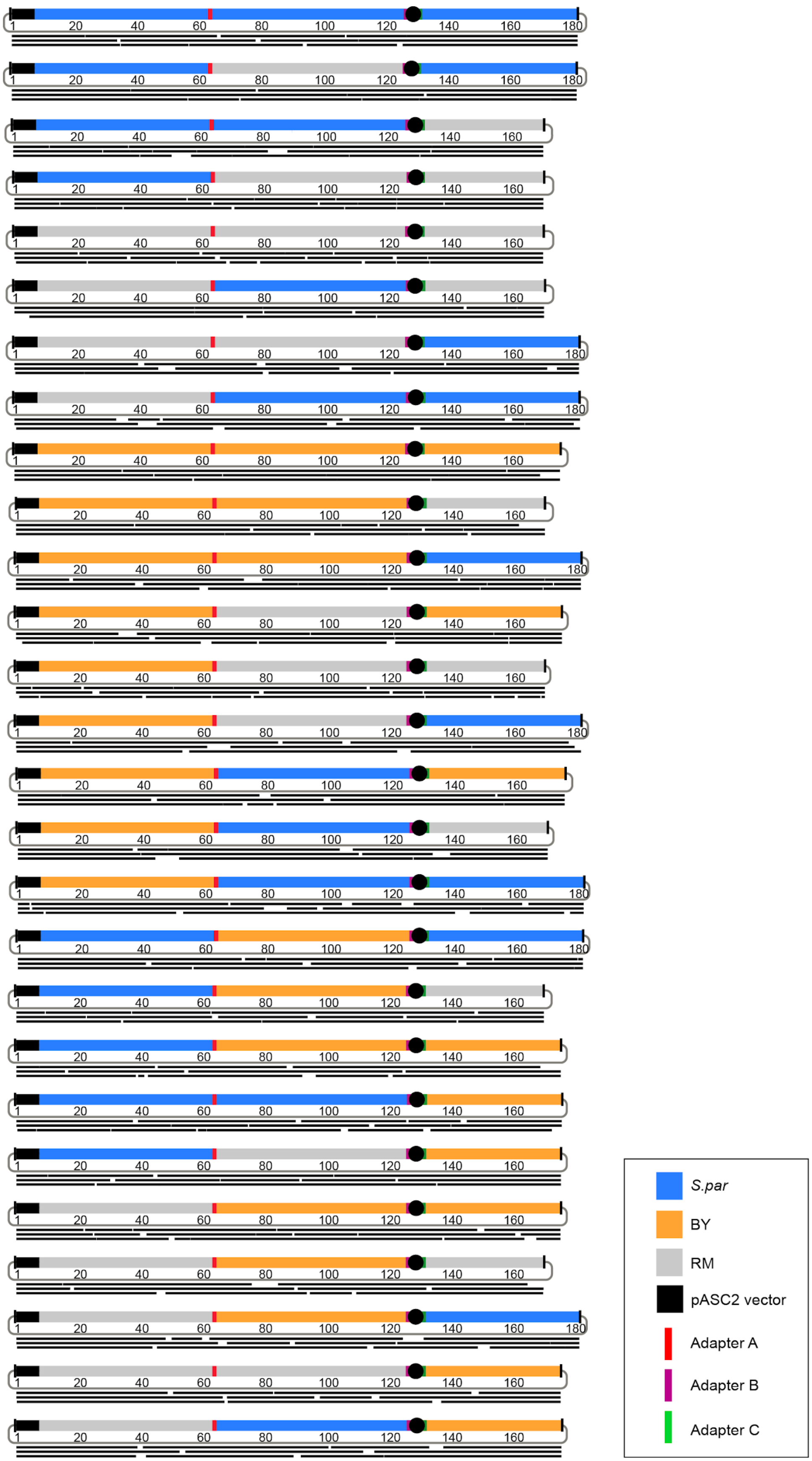
S.par
BY
RM
pASC2 vector
Adapter A
Adapter B
Adapter C

Supplementary Figure 9: Oxford Nanopore Technologies (ONT) long-read sequencing confirms the structures of 24 recombinant versions of ChrI. Each recombinant version of ChrI represented here contains a region corresponding to the three assembled segments (colored according to their donor), the adapters present in the assembly junctions, and the centromere cassette (black circle). Below each chimeric chromosome we have aligned ONT reads covering the entire chromosome, confirming correct assembly.

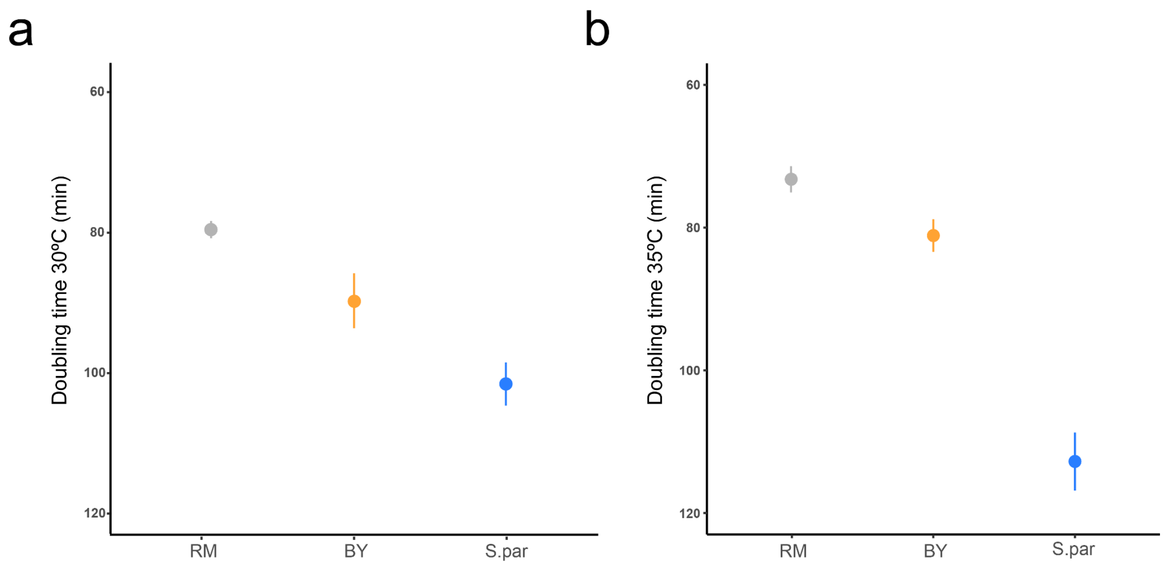

Supplementary Figure 10: Donor strains show growth differences at 30ºC and 35ºC. The plot shows the mean and standard deviation of doubling time for RM (gray), BY (orange), and *S.paradoxus* (blue) grown at 30ºC (**a**) and 35ºC (**b**). A total of 9 replicates were phenotyped per strain. The y-axis scale is inverted.

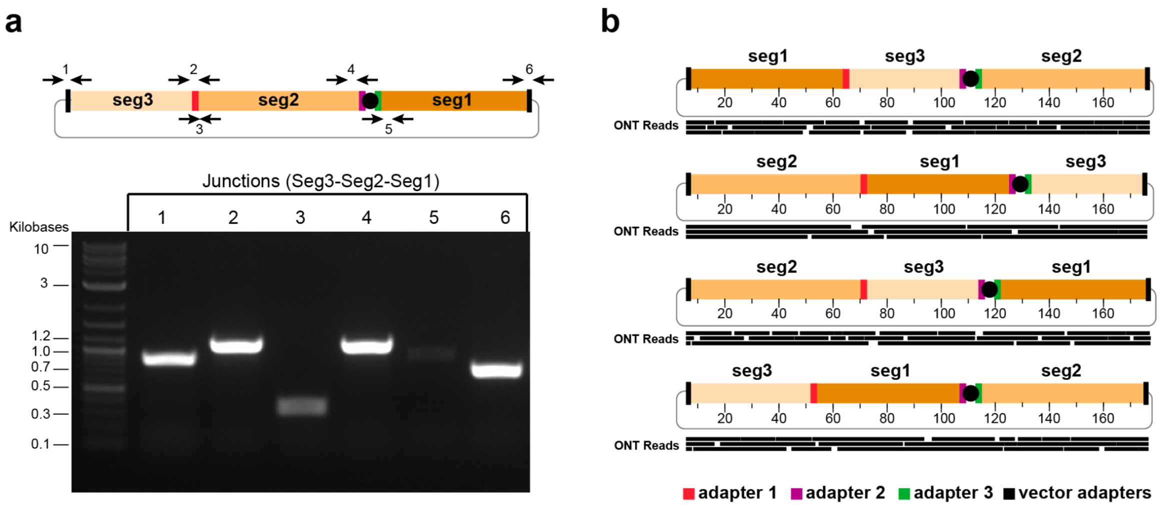

Supplementary Figure 11: Assembly of restructured versions of BY ChrI. a. The correct assembly of each restructured version of ChrI was initially checked by junction PCRs as shown for restructuring 3-2-1**.** Each pair of arrows represent specific designed junction primers. The 1% gel shows the correct amplicons for each analyzed junction on restructuring 3-2-1 (IEY424). All 4 remaining restructured ChrI versions are represented. Each contains a region corresponding to the three assembled segments (colored according to their original position on parental ChrI), the adapters present in the assembly junctions, and the centromere cassette (black circle). Below each restructured chromosome we have aligned ONT reads covering the entire chromosome, confirming correct assembly.

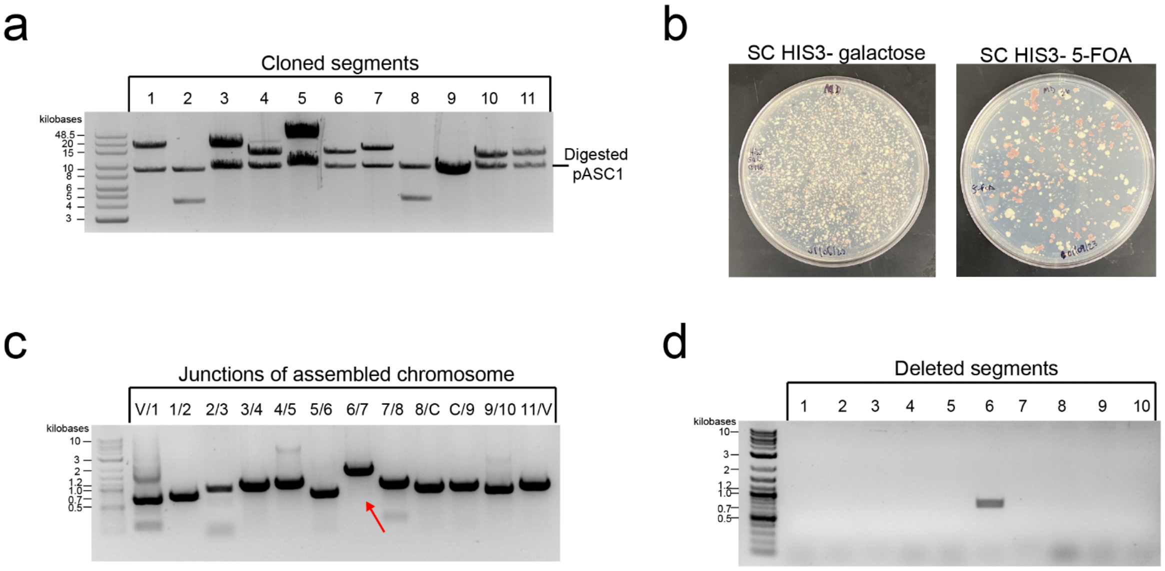

Supplementary Figure 12: PCR checks confirm that CReATiNG deleted nine core chromosome regions and that *SYN8* remained. a. The 11 cloned segments adjacent to the target elements were digested using I-SceI enzyme and ran for 90 mins on an 0.5% agarose gel to allow separation of the cloning vector (pASC1) and cloned segments. For each well (1-11), a double DNA band is expected showing separation of the segment and vector. Well 9 shows a single band at pASC1 size but the cloned segment has roughly the same size of pASC1 (8.4 vs. 9.1kb), making it difficult to distinguish the bands. **b.** Plate image of multiple deletion transformants growing in SC galactose plates lacking histidine (left) and SC 5-FOA plates lacking histidine (right). Presence of pink colonies in both plates is an indicator of *ADE1* deletion and was used as an additional check for chromosome replacement. **c.** Junction PCRs were performed to confirm correct assembly of the multiple deletion ChrI. Reactions were checked by 1% agarose gel electrophoresis. Expected products were seen for all junctions except between segment 6 and 7. This final check was larger than expected (by 845 bp), suggesting the region targeted for deletion, which contains *SYN8*, was retained. Red arrow points to the expected DNA size for successful deletion of the *SYN8* region. Persistence of the *SYN8* region was confirmed by region-specific PCR reactions for all deleted regions, as shown in **d.**

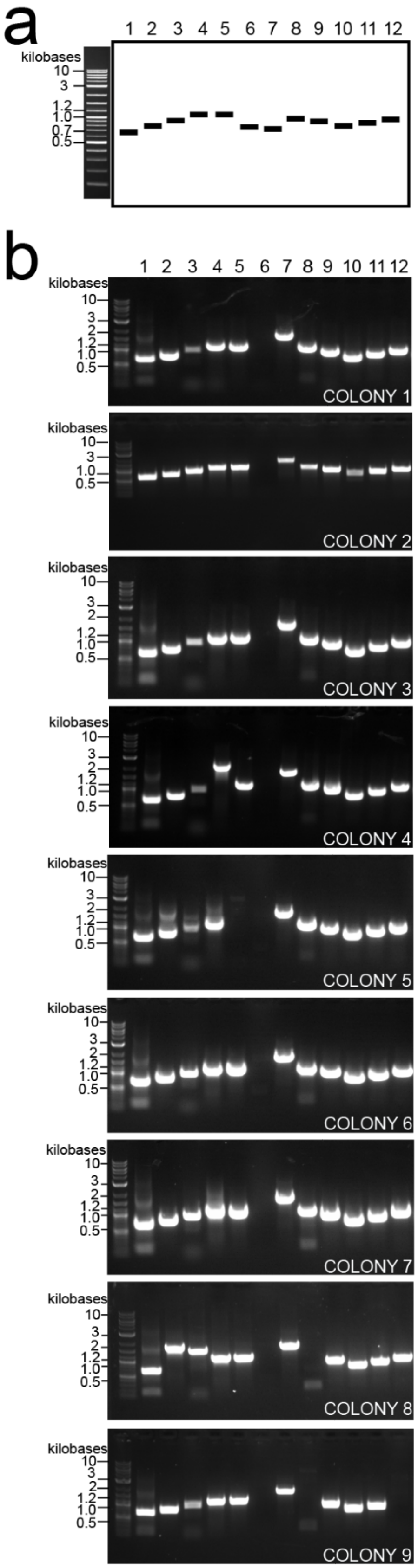

Supplementary Figure 13: Additional colonies checked for correct assembly of multiple deletion ChrI. a. Scheme representing expected amplicon size for all 12 junction PCRs on multiple deletion ChrI. Expected sizes are: 1=664bp; 2=782bp; 3=947bp; 4=1140bp; 5=1150bp; 6=777bp; 7=736bp; 8=921bp; 9= 897bp; 10=766bp; 11=834bp; 12=1024bp. **b.** Junction PCR reactions were checked by electrophoresis with a 1% agarose gel. All colonies did not show the expected amplicon size for junction 7, which contains *SYN8*. Instead the observed size corresponds to retention of *SYN8*, suggesting selective pressure for maintenance of this gene.

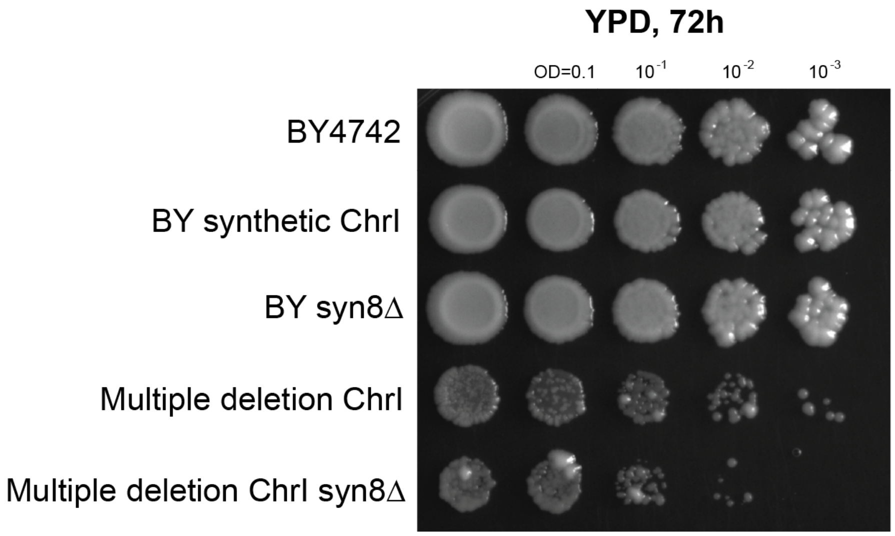

Supplementary Figure 14: Dilution assays confirm the slow growth of ChrI multiple deletion strains with and without *SYN8*.

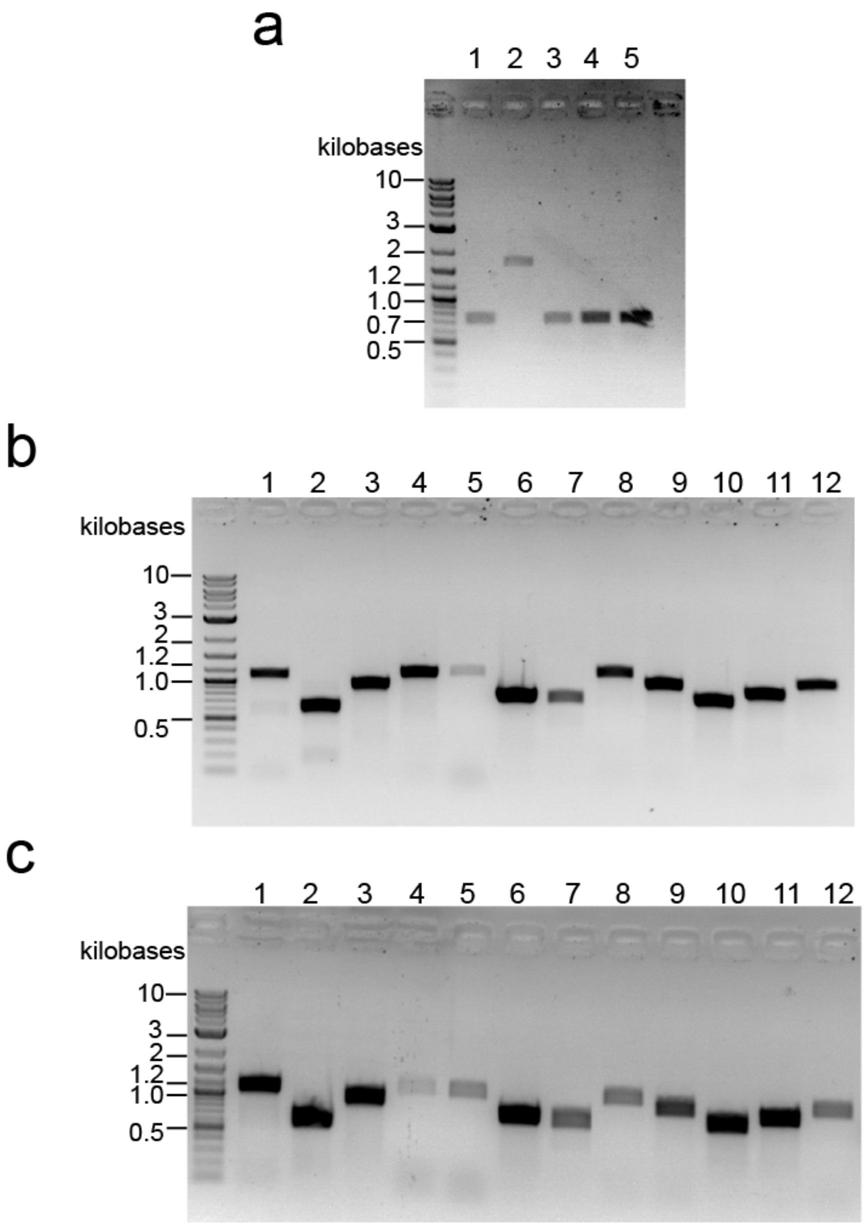

Supplementary Figure 15: PCR checking of colonies from the additional multiplex deletion experiment individually submitted to chromosome elimination. a. We checked five colonies growing on the transformation plate for presence of synthetic junctions between segment 6 and 7 and four colonies showed the expected amplicon size (756 bp). One of these 4 colonies had all the other synthetic junctions confirmed by PCR before (**b**) and after native chromosome elimination (**c**). Expected sizes are: 1 = 1.162 bp; 2 = 782 bp; 3 = 947 bp; 4 = 1140 bp; 5 = 1150 bp; 6 = 777 bp; 7 = 736 bp; 8 = 921 bp; 9 = 897 bp; 10 = 766 bp; 11 = 834 bp; 12 = 1024 bp.

Vector	Addgene link
Capture Vector (pASC1)	HTTPS://WWW.ADDGENE.ORG/209412/
Assembly vector (pASC2)	HTTPS://WWW.ADDGENE.ORG/209413/
pML104 *HIS3*	HTTPS://WWW.ADDGENE.ORG/209957/
pRS316 *NatMX*	HTTPS://WWW.ADDGENE.ORG/210014/

Supplementary Table 1: Link for genbank files of vectors pASC1, pASC2, pML104 *HIS3*, and pRS316 created during development of this study.

Strain	Genotype	Description
S.cerevisiae BY4742	MATα his3Δ1 leu2Δ0 lys2Δ0 ura3Δ0	Deletion strains derived from lab strain S288C
S.cerevisiae RM11-1a	MATa leu2Δ0 ura3-Δ0 HO::kanMX	Natural isolate from California vineyards
S.paradoxus CBS5829	MATa URA3 his3Δ::cloNatMX	Natural isolate from soil (Denmark)
IEY394	MATα leu2Δ0 lys2Δ0 ura3Δ0	BY4742 derivative containing a chimeric ChrI with all three segments from *S.paradoxus*
IEY395	MATα leu2Δ0 lys2Δ0 ura3Δ0	BY4742 derivative containing a chimeric ChrI with segments 1, 2, and 3 from *S.cer* RM, *S.par*, and *S.par*, respectively.
IEY396	MATα leu2Δ0 lys2Δ0 ura3Δ0	BY4742 derivative containing a chimeric ChrI with segments 1, 2, and 3 derived from *S.par*, *S.cer* RM, and *S.par*, respectively.
IEY397	MATα leu2Δ0 lys2Δ0 ura3Δ0	BY4742 derivative containing a chimeric ChrI with segments 1, 2, and 3 from *S.par*, *S.par*, and *S.cer* RM, respectively.
IEY398	MATα leu2Δ0 lys2Δ0 ura3Δ0	BY4742 derivative containing a chimeric ChrI with segments 1, 2, and 3 from *S.cer* RM, *S.cer* RM, and *S.cer* RM, respectively.
IEY399	MATα leu2Δ0 lys2Δ0 ura3Δ0	BY4742 derivative containing a chimeric ChrI with segments 1, 2, and 3 from *S.par*, *S.cer* RM, and *S.cer* RM, respectively.
IEY400	MATα leu2Δ0 lys2Δ0 ura3Δ0	BY4742 derivative containing a chimeric ChrI with segments 1, 2, and 3 from *S.cer* RM, *S.par*, and *S.cer* RM, respectively.
IEY401	MATα leu2Δ0 lys2Δ0 ura3Δ0	BY4742 derivative containing a chimeric ChrI with segments 1, 2, and 3 from *S.cer* RM, *S.cer* RM, and *S.par*, respectively.

IEY402	MATα leu2Δ0 lys2Δ0 ura3Δ0	BY4742 derivative containing a chimeric ChrI with segments 1, 2, and 3 from *S.cer* BY, *S.cer* BY, and *S.cer* BY, respectively.
IEY403	MATα leu2Δ0 lys2Δ0 ura3Δ0	BY4742 derivative containing a chimeric ChrI with segments 1, 2, and 3 from *S.cer* BY, *S.cer* BY, and *S.cer* RM, respectively.
IEY404	MATα leu2Δ0 lys2Δ0 ura3Δ0	BY4742 derivative containing a chimeric ChrI with segments 1, 2, and 3 from *S.cer* BY, *S.cer* BY, and *S.par*, respectively.
IEY405	MATα leu2Δ0 lys2Δ0 ura3Δ0	BY4742 derivative containing a chimeric ChrI with segments 1, 2, and 3 from *S.cer* BY, *S.cer* RM, and *S.cer* BY, respectively.
IEY406	MATα leu2Δ0 lys2Δ0 ura3Δ0	BY4742 derivative containing a chimeric ChrI with segments 1, 2, and 3 from *S.cer* BY, *S.cer* RM, and *S.cer* RM, respectively.
IEY407	MATα leu2Δ0 lys2Δ0 ura3Δ0	BY4742 derivative containing a chimeric ChrI with segments 1, 2, and 3 from *S.cer* BY, *S.cer* RM, and *S.par*, respectively.
IEY408	MATα leu2Δ0 lys2Δ0 ura3Δ0	BY4742 derivative containing a chimeric ChrI with segments 1, 2, and 3 from *S.cer* BY, *S.par*, and *S.cer* BY, respectively.
IEY409	MATα leu2Δ0 lys2Δ0 ura3Δ0	BY4742 derivative containing a chimeric ChrI with segments 1, 2, and 3 from *S.cer* BY, *S.par*, and *S.cer* RM, respectively.
IEY410	MATα leu2Δ0 lys2Δ0 ura3Δ0	BY4742 derivative containing a chimeric ChrI with segments 1, 2, and 3 from *S.cer* BY, *S.par*, and *S.par*, respectively.
IEY411	MATα leu2Δ0 lys2Δ0 ura3Δ0	BY4742 derivative containing a chimeric ChrI with segments 1, 2, and 3 from *S.par*, *S.cer* BY, and *S.par*, respectively.
IEY412	MATα leu2Δ0 lys2Δ0 ura3Δ0	BY4742 derivative containing a chimeric ChrI with segments 1, 2, and 3 from *S.par*, *S.cer* BY, and *S.cer* RM, respectively.
IEY413	MATα leu2Δ0 lys2Δ0 ura3Δ0	BY4742 derivative containing a chimeric ChrI with segments 1, 2, and 3 from *S.par*, *S.cer* BY, and *S.cer* BY, respectively.

IEY414	MATα leu2Δ0 lys2Δ0 ura3Δ0	BY4742 derivative containing a chimeric ChrI with segments 1, 2, and 3 from *S.par*, *S.par*, and *S.cer* BY, respectively.
IEY415	MATα leu2Δ0 lys2Δ0 ura3Δ0	BY4742 derivative containing a chimeric ChrI with segments 1, 2, and 3 from *S.par*, *S.cer* RM, and *S.cer* BY, respectively.
IEY416	MATα leu2Δ0 lys2Δ0 ura3Δ0	BY4742 derivative containing a chimeric ChrI with segments 1, 2, and 3 from *S.cer* RM, *S.cer* BY, and *S.cer* BY, respectively.
IEY417	MATα leu2Δ0 lys2Δ0 ura3Δ0	BY4742 derivative containing a chimeric ChrI with segments 1, 2, and 3 from *S.cer* RM, *S.cer* BY, and *S.cer* RM, respectively.
IEY418	MATα leu2Δ0 lys2Δ0 ura3Δ0	BY4742 derivative containing a chimeric ChrI with segments 1, 2, and 3 from *S.cer* RM, *S.cer* BY, and *S.par*, respectively.
IEY419	MATα leu2Δ0 lys2Δ0 ura3Δ0	BY4742 derivative containing a chimeric ChrI with segments 1, 2, and 3 from *S.cer* RM, *S.cer* RM, and *S.cer* BY, respectively.
IEY420	MATα leu2Δ0 lys2Δ0 ura3Δ0	BY4742 derivative containing a chimeric ChrI with segments 1, 2, and 3 from *S.cer* RM, *S.par*, and *S.cer* BY, respectively.
IEY421	MATα leu2Δ0 lys2Δ0 ura3Δ0	BY4742 derivative containing a restructured ChrI with segments 1,2 and 3 rearranged as 1-3-2.
IEY422	MATα leu2Δ0 lys2Δ0 ura3Δ0	BY4742 derivative containing a restructured ChrI with segments 1,2 and 3 rearranged as 2-1-3.
IEY423	MATα leu2Δ0 lys2Δ0 ura3Δ0	BY4742 derivative containing a restructured ChrI with segments 1,2 and 3 rearranged as 2-3-1.
IEY424	MATα leu2Δ0 lys2Δ0 ura3Δ0	BY4742 derivative containing a restructured ChrI with segments 1,2 and 3 rearranged as 3-2-1.
IEY425	MATα leu2Δ0 lys2Δ0 ura3Δ0	BY4742 derivative containing a restructured ChrI with segments 1,2 and 3 rearranged as 3-1-2.

IEY426	MATα his3Δ1 leu2Δ0 lys2Δ0 syn8::NatMX	BY4742 derivative containing a SYN8 gene full replacement by *NatMX* drug resistance cassette
IEY427	MATα leu2Δ0 lys2Δ0 ura3Δ0	BY4742 derivative containing all the intended deletions across ChrI but not SYN8. A list with all the deleted genes can be found on Supplementary Table 17 and 20.
IEY428	MATα leu2Δ0 lys2Δ0 ura3Δ0 syn8::NatMX	IEY427 derivate strain containing SYN8 gene full replacement by *NatMX* drug resistance cassette
IEY429	MATα his3Δ1 leu2Δ0 lys2Δ0 CEN1::Ura3-pGal1-CEN1	BY4742 derivative containing a conditional centromere and used as recipient cell in all CReATiNG assembly experiments

Supplementary Table 2: Yeast strain table containing all the strains used and generated in this study. Column 1 ('Strain') lists the ID of all yeast strains. Column 2 ('Genotype') lists the genotype of each strain. Column 3 (‘Description’) brings a brief description about origin and genetic modifications of each strain.

Cloning cassette	Sequence
BY or RM segment 1	TATTTTACGCGCAAGCCGGACCCGCGCTTGCACGGCTGAGGTGCGTAGG<br>GAACGTCGCAAAGAGGCATGAATATTCAGCAGCACAGAACAAATTAATCA<br>CGAGCCAATGAGATTTTAAAGGGTATATTACTTATCTTATCGATAAGCAGTA<br>TTGATATTAAAGGGACAGTTTTATCGTTGGTTAATATGGAAAAAGTGATGAC<br>CATGATGCCTTTCTTAAAAAGAGTATTTCTTTTTATTTCACTTTCACCTCGA<br>GAGTCCTAGGTATTTTTAGGAAATTGATGATACCGTGGGAATAATGAGAGA<br>TAACATAAATAAAGTAGCAGAAAGAGGTGAAAGATTAACGTCCATTGAAGA<br>TAAAGCCGATAACCTAGCGGTCTCAGCCCAAGGCTTTAAGAGGGGTGCC<br>AATAGGGTCCTTACATATCCTCTACACCGAGCGCGTCGACCCGTCGAATG<br>AATGTGAGTTGCGTGTTGTTCCTTCGGAAATGTTGCTAAAAAACAGAGCC<br>ATTCCTTTT
BY or RM segment 2	CTTACATATCCTCTACACCGAGCGCGTCGACCCGTCGAATGAATGTGAGT<br>TGCGTGTTGTTCCTTCGGAAATGTTGCTAAAAAACAGAGCCATTCCTTTTT<br>ATATGTATATATACATATATACATATATGGGCGTATATTTACTTTGTTCTTATTT<br>TCTGTCTAATTTTATAATTTTACTGACAGTAGCTAAGCCCTCTGTATTGCTG<br>TTCTGTGTTATTGCACTAGTGTCATAACGCAGATGGTTTTTACTCGAGAGT<br>CCTAGCTATTTGTACGAGTTCGTCAGGATAAATCGTCAGTACCATTTTTCTT<br>GTGGCTAGTTGGCTTCAACCAAACGTCCTCTTCTCTCTTATGGCAAGAAG<br>AAAGTTATATGTGTGACTGGTTGTTTATTTCACTTTCGCGACTGAAAGCGC<br>CGTCTAAACTAGAAAGTTACCGTTAGTCTTACTGATCCGAACCGTCATTAG<br>TTACTAGAGGAAACGTGTCAAGAATGCCAGCCGATCCCACATTCTTAATTA
BY or RM segment 3	AATCAAAGGGAAGTCGAATAAAGGTCATCCAGGGAATTAGCCGTTTTCGA<br>CCAAGTCTTTACGGGAGTAGAAGACGAATCCTCTCGTGTGCTGGCCCAG<br>GAAATTAAGATAGTTATTCGAGTGATTGCCAAATATCATGTTCTACTTCGAA<br>GACTTATAGCTAATTAATTTTTTCATAATGAAGGTGTCGTTAATTGTTCTGAT<br>TAGTAACATGAAACTCAAAAATCATCAAAAAAAGAAAAGCTAAATGCTCGA<br>GAGTCCTAGGAAAGGATCATGGCTGGCAGCGCCCACAACAATTAAACTCT<br>GTTACTATCAAAAAACATTGAGCCCAAGAATGGAATAAAATTTTCACTACAC<br>CTCGGACATGGATTTGTACATGTCCTATTATCCTGTAATTTTGACATATACT<br>GATATGACTTGATACCTAGTTAACCCACGGGTGTGGAAAAGCTCCCTAAG<br>GGAGGAACGGACGCCGGAAGGTTTTCCTGAGTGGCACTCACACATTATG<br>GTGTCAG
S.paradoxus segment 1	TATTTTACGCGCAAGCCGGACCCGCGCTTGCACGGCTGAGGTGCGTAGG<br>GAACGTCGCAAAGAGGCATGAATATTCAGCAGCACAGAACAAATTAATCA<br>CTAGAAACGGCTGACAACCATTTTGTTAGTCCTCGGAAAATCTCCGAACA<br>AATGAGAAAGCAAAATATAATAACACAGAGTTTGGCGAGAAGAAGATATAT<br>GATAACTACTATTAAGGACCCTCGAGAGTCCTAGGTAGAAACGGCTGACA<br>ACCATTTTGTTAGTCCTCGGAAAATCTCCGAACAAATGAGAAAGCAAAATA<br>TAATAACACAGAGTTTGGCGAGAAGAAGATATATGATAACTACTATTAAGGA<br>CCCTTACATATCCTCTACACCGAGCGCGTCGACCCGTCGAATGAATGTGA<br>GTTGCGTGTTGTTCCTTCGGAAATGTTGCTAAAAAACAGAGCCATTCCTTT<br>T
S.paradoxus segment 2	CTTACATATCCTCTACACCGAGCGCGTCGACCCGTCGAATGAATGTGAGT<br>TGCGTGTTGTTCCTTCGGAAATGTTGCTAAAAAACAGAGCCATTCCTTTTT<br>ATATATGAGAGCGTATATGTACTTTGTTGTTTTTATTTTTTCCTGCTTAGTTC<br>TATAATCTTATTGACATTTGTCAAGCCCTCTGTATTGCAGTTATGTGGTATT<br>GCACCGGTGTCATACTCGAGAGTCCTAGGTCACATGTTACTTTTATAAAAT

	AATTATATTATATTTTAAATATATCAAAGTTTCTTTCATTTTTTAGATTACTTTA<br>AGATAATAAATATATGTTTTTGGTTTCCGAAACGTTTAAAATATCTAAACTAG<br>AAAGTTACCGTTAGTCTTACTGATCCGAACCGTCATTAGTTACTAGAGGAA<br>ACGTGTCAAGAATGCCAGCCGATCCCACATTCTTAATTA
S.paradoxus segment 3	AATCAAAGGGAAGTCGAATAAAGGTCATCCAGGGAATTAGCCGTTTTCGA<br>CCAAGTCTTTACGGGAGTAGAAGACGAATCCTCTCGTGTGCTGGCCCAG<br>GCCAGCGATAGCCCATTGTGTAATGTGTATTTCTAACCATCAAACACTATAC<br>CTCTACGCCAAATAGACAACGCAAGACACTCTATAAACGGAAACCCAATG<br>CACCATAAATCATTAAGGACTCGAGAGTCCTAGGCCAGCGATAGCCCATT<br>GTGTAATGTGTATTTCTAACCATCAAACACTATACCTCTACGCCAAATAGAC<br>AACGCAAGACACTCTATAAACGGAAACCCAATGCACCATAAATCATTAAGG<br>AACTTGATACCTAGTTAACCCACGGGTGTGGAAAAGCTCCCTAAGGGAGG<br>AACGGACGCCGGAAGGTTTTCCTGAGTGGCACTCACACATTATGGTGTC<br>AG
Centromere cassette	TCTAAACTAGAAAGTTACCGTTAGTCTTACTGATCCGAACCGTCATTAGTTA<br>CTAGAGGAAACGTGTCAAGAATGCCAGCCGATCCCACATTCTTAATTACT<br>CAGTGGAACGAAAACTCACGTTAAGGGATTTTGGTCATGAGATTATCAAAA<br>AGGATCTTCACCTAGATCCTTTTAAATTAAAAATGAAGTTTTAAATCAATCTA<br>AAGTATATATGAGTAAACTTGGTCTGACAGTTACCAATGCTTAATCAGTGAG<br>GCACCTATCTCAGCGATCTGTCTATTTCGTTCATCCATAGTTGCCTGACTG<br>CCCGTCGTGTAGATAACTACGATACGGGAGGGCTTACCATCTGGCCCCAG<br>TGCTGCAATGATACCGCGAGACCCACGCTCACCGGCTCCAGATTTATCAG<br>CAATAAACCAGCCAGCCGGAAGGGCCGAGCGCAGAAGTGGTCCTGCAA<br>CTTTATCCGCCTCCATCCAGTCTATTAATTGTTGCCGGGAAGCTAGAGTAA<br>GTAGTTCGCCAGTTAATAGTTTGCGCAACGTTGTTGCCATTGCTACAGGC<br>ATCGTGGTGTCACGCTCGTCGTTTGGTATGGCTTCATTCAGCTCCGGTTC<br>CCAACGATCAAGGCGAGTTACATGATCCCCCATGTTGTGAAAAAAAGCGG<br>TTAGCTCCTTCGGTCCTCCGATCGTTGTCAGAAGTAAGTTGGCCGCAGTG<br>TTATCACTCATGGTTATGGCAGCACTGCATAATTCTCTTACTGTCATGCCAT<br>CCGTAAGATGCTTTTCTGTGACTGGTGAGTACTCAACCAAGTCATTCTGA<br>GAATAGTGTATGCGGCGACCGAGTTGCTCTTGCCCGGCGTCAATACGGG<br>ATAATACCGCGCCACATAGCAGAACTTTAAAAGTGCTCATCATTGGAAAAC<br>GTTCTTCGGGGCGAAAACTCTCAAGGATCTTACCGCTGTTGAGATCCAGT<br>TCGATGTAACCCACTCGTGCACCCAACTGATCTTCAGCATCTTTTACTTTC<br>ACCAGCGTTTCTGGGTGAGCAAAAACAGGAAGGCAAAATGCCGCAAAAA<br>AGGGAATAAGGGCGACACGGAAATGTTGAATACTCATACTCTTCCTTTTTC<br>AATATTATTGAAGCATTTATCAGGGTTATTGTCTCATGAGCGGATACATATTT<br>GAATGTATTTAGAAAAATAAACAAATAGGGGTTCCGCGCACATTTCCCCGA<br>AAAGTGCCACCTGGGTCCTTTTCATCACGTGCTATAAAAATAATTATAATTT<br>AAATTTTTTAATATAAATATATAAATTAAAAATAGAAAGTAAAAAAAGAAATTA<br>AAGAAAAAATAGTTTTTGTTTTCCGAAGATGTAAAAGACTCTAGGGGGATC<br>GCCAACAAATACTACCTTTTATCTTGCTCTTCCTGCTCTCAGGTATTAATGC<br>CGAATTGTTTCATCTTGTCTGTGTAGAAGACCACACACGAAAATCCTGTG<br>ATTTTACATTTTACTTATCGTTAATCGAATGTATATCTATTTAATCTGCTTTTC<br>TTGTCTAATAAATATATATGTAAAGTACGCTTTTTGTTGAAATTTTTTAAACCT<br>TTGTTTATTTTTTTTTCTTCATTCCGTAACTCTTCTACCTTCTTTATTTACTTT<br>CTAAAATCCAAATACAAAACATAAAAATAAATAAACACAGAGTAAATTCCCA<br>AATTATTCCATCATTAAAAGATACGAGGCGCGTGTAAGTTACAGGCAAGCG<br>ATCCGTCCTAAGAAACCATGACGAGTACGGTGGGTAGGCCGGCCAGCGA<br>CATGGAGGCCCAGAATACCCTCCTTGACAGTCTTGACGTGCGCAGCTCA<br>GGGGCATGATGTGACTGTCGCCCGTACATTTAGCCCATACATCCCCATGT<br>ATAATCATTTGCATCCATACATTTTGATGGCCGCACGGCGCGAAGCAAAAA<br>TTACGGCTCCTCGCTGCAGACCTGCGAGCAGGGAAACGCTCCCCTCACA

	GACGCGTTGAATTGTCCCCACGCCGCGCCCCTGTAGAGAAATATAAAAG<br>GTTAGGATTTGCCACTGAGGTTCTTCTTTCATATACTTCCTTTTAAAATCTT<br>GCTAGGATACAGTTCTCACATCACATCCGAACATAAACAACCATGGGTAAG<br>GAAAAGACTCACGTTTCGAGGCCGCGATTAAATTCCAACATGGATGCTGA<br>TTTATATGGGTATAAATGGGCTCGCGATAATGTCGGGCAATCAGGTGCGAC<br>AATCTATCGATTGTATGGGAAGCCCGATGCGCCAGAGTTGTTTCTGAAAC<br>ATGGCAAAGGTAGCGTTGCCAATGATGTTACAGATGAGATGGTCAGACTA<br>AACTGGCTGACGGAATTTATGCCTCTTCCGACCATCAAGCATTTTATCCGT<br>ACTCCTGATGATGCATGGTTACTCACCACTGCGATCCCCGGCAAAACAGC<br>ATTCCAGGTATTAGAAGAATATCCTGATTCAGGTGAAAATATTGTTGATGCG<br>CTGGCAGTGTTCCTGCGCCGGTTGCATTCGATTCCTGTTTGTAATTGTCC<br>TTTTAACAGCGATCGCGTATTTCGTCTCGCTCAGGCGCAATCACGAATGA<br>ATAACGGTTTGGTTGATGCGAGTGATTTTGATGACGAGCGTAATGGCTGG<br>CCTGTTGAACAAGTCTGGAAAGAAATGCATAAGCTTTTGCCATTCTCACC<br>GGATTCAGTCGTCACTCATGGTGATTTCTCACTTGATAACCTTATTTTTGA<br>CGAGGGGAAATTAATAGGTTGTATTGATGTTGGACGAGTCGGAATCGCAG<br>ACCGATACCAGGATCTTGCCATCCTATGGAACTGCCTCGGTGAGTTTTCT<br>CCTTCATTACAGAAACGGCTTTTTCAAAAATATGGTATTGATAATCCTGATA<br>TGAATAAATTGCAGTTTCATTTGATGCTCGATGAGTTTTTCTAATCAGTACT<br>GACAATAAAAAGATTCTTGTTTTCAAGAACTTGTCATTTGTATAGTTTTTTTA<br>TATTGTAGTTGTTCTATTTTAATCAAATGTTAGCGTGATTTATATTTTTTTTCG<br>CCTCGACATCATCTGCCCAGATGCGAAGTTAAGTGCGCAGAAAGTAATAT<br>CATGCGTCAATCGTATGTGAATGCTGGTCGCTATACTGCTGTCGATTCGAT<br>ACTAACGCCGCCATCCAGTGTCGAAAACTGCTTGGCAACCTAATGCTANN<br>ANNANNANNANNANNANNANNANNANNATACCGTTCGTATAATGTGTACTA<br>TACGAAGTTATTCAATCGGTGTCACTTATGGATAACTTCGTATAATGTATGC<br>TATACGAACGGTAAATCAAAGGGAAGTCGAATAAAGGTCATCCAGGGAAT<br>TAGCCGTTTTCGACCAAGTCTTTACGGGAGTAGAAGACGAATCCTCTCGT<br>GTGCTGGCCCAGG

Upstream adapter; upstream homology arm; downstream homology; and downstream adapter

Supplementary Table 3: Cloning cassettes for segments 1 through 3 in all experiments except multiplex deletion. Each cassette contains a pair of upstream and downstream adapters flanking segment-specific homology arms separated by XhoI and AvrII sites used for vector linearization. The same cloning cassettes were used in BY and RM, while different cloning cassettes were used for *S.paradoxus*.

gRNAs	Sequence
S.cerevisiae S1 gRNA1 up	CATTAAAAAGAAGGCATAGG
S.cerevisiae S1 gRNA2 up	TTTGAAGAAAGGAAAAAATG
S.cerevisiae S1 gRNA3 up	GGCCAATCAAGCAAACTGAA
S.cerevisiae S1 gRNA1 down	TCTAAAAATGAAGATGTGTC
S.cerevisiae S1 gRNA2 down	ACACAGAACAGCAATACAGA
S.cerevisiae S1 gRNA3 down	AACACAGAACAGCAATACAG
S.cerevisiae S2 gRNA1 up	TTTTAGATCCTTGTACCACA
S.cerevisiae S2 gRNA2 up	TCTAAAAATGAAGATGTGTC
S.cerevisiae S2 gRNA1 down	CTGCATTTTCAAATACCGCT
S.cerevisiae S2 gRNA2 down	GTTTTTTGCATCATAAAGGG
S.cerevisiae S2 gRNA3 down	TCAATATAATAAGCATACTA
S.cerevisiae S3 gRNA1 up	CACCTTTCGTCTGCTGCCTC
S.cerevisiae S3 gRNA2 up	CACCCGAGGCAGCAGACGAA
S.cerevisiae S3 gRNA3 up	ATAATTTGAAAAAACACCCG
S.cerevisiae S3 gRNA1 down	ATCCTTTTTTTATTTGAAAG
S.cerevisiae S3 gRNA2 down	AATAAAAAAAGGATCATGGC
S.cerevisiae S3 gRNA3 down	AAAAACATTGAGCCCAAGAA
S.paradoxus S1 gRNA1 up	CATTATAAAAATTAAAACCG
S.paradoxus S1 gRNA2 up	GATTAACGGAGCTGCCCAAG
S.paradoxus S1 gRNA3 up	ATGGAAACCTCCTTCTGTGG
S.paradoxus S1 gRNA1 down	CAATGTTTAACTAATACTCT
S.paradoxus S1 gRNA2 down	TCTCTGCTCAAGGTTTCAAG
S.paradoxus S1 gRNA3 down	ATAGGGTGAGGAAAGCCATG
S.paradoxus S2 gRNA1 up	TTAGTCGATAGAAATTCACT

S.paradoxus S2 gRNA2 up	GAATACTATATATATATAGG
S.paradoxus S2 gRNA3 up	ATAAGATTATAGAACTAAGC
S.paradoxus S2 gRNA1 down	ATACCCGTCCAGAATGTGAG
S.paradoxus S2 gRNA2 down	TACCCGTCCAGAATGTGAGA
S.paradoxus S2 gRNA3 down	ATAGATTTTCTGACCAGTAG
S.paradoxus S3 gRNA1 up	CATAACTAACTCGATTCGCA
S.paradoxus S3 gRNA2 up	ATGGATTGCCAACGATGATG
S.paradoxus S3 gRNA3 up	ACTGAGAGCCAAGAATTCAA
S.paradoxus S3 gRNA1 down	AAAGTTGAGATATTCTGGAT
S.paradoxus S3 gRNA2 down	CAAACTTCATGCAGGATGCT
S.paradoxus S3 gRNA3 down	AATTTCGGAAAAAAAGTCCC

Supplementary Table 4: gRNAs used to clone the three segments in the initial chromosome substitution, synthetic recombination, and restructuring experiments. Column 1 (‘gRNAs’) lists the gRNAs IDs. The annotations S1, S2 and S3 represent segment 1, 2 and 3, respectively. The ‘up’ and ‘down’ annotations refer to the side of a segment targeted by a guide. Column 2 (‘sequence’) lists the target sequence of each gRNA.

Primers	Sequence
S.cerevisiae S1 clone check REV	GGAAGCGTAGGCCACCCT
S.cerevisiae S1 clone check FOR	TCCGAGCACGATGAAGAACGA
S.cerevisiae S2 clone check REV	AAGCCTGCTAACAAGGGCGA
S.cerevisiae S2 clone check FOR	CCAGTTACCTGCGCCAAATC
S.cerevisiae S3 clone check REV	CTGGCTTCACCCACTGCAGA
S.cerevisiae S3 clone check FOR	CCATGAGCTGCGTGGCATT
S.paradoxus S1 clone check REV	CCGATGGGTGAAAGCGTAGC
S.paradoxus S1 clone check FOR	GAACGGCCTCAGAATGTGCAG
S.paradoxus S2 clone check REV	GAGGCGAAGCTGGAGTACCG
S.paradoxus S2 clone check FOR	AAGCGCCCCTTCTGATGCA
S.paradoxus S3 clone check REV	AACAGTGACACCACACCCTCT
S.paradoxus S3 clone check FOR	CTTGTTGGCGTGTGAGGAGG
Centromere check REV	AAAGTTGCAGGACCACTTCTGC
Centromere check FRW	CCCAGATGCGAAGTTAAGTG
pASC1 check FRW	CAAGGGAGACGCATTGGGTCA
pASC1 check REV	GCCTGGTTGCTACGCCTGA

Supplementary Table 5: Primers used to confirm cloning of segments 1 through 3 for all experiments except multiplex deletion. Column 1 (‘Primers’) lists primers IDs. Column 2 (‘Sequence’) contains the nucleotide sequence of each primer.

**Cloned segment**	**Size (kb)**	**Upstream junction check**	**Downstream junction check**	**Cloning efficiency**
S.par 1	56.6	5/5 (100%)	5/5 (100%)	100%
S.par 2	63.9	5/5 (100%)	4/5 (80%)	80%
S.par 3	53.9	5/5 (100%)	5/5 (100%)	100%
BY 1	56.5	5/5 (100%)	5/5 (100%)	100%
BY 2	63.4	4/5 (80%)	4/5 (80%)	80%
BY 3	45.1	5/5 (100%)	5/5 (100%)	100%
RM 1	56.5	5/5 (100%)	5/5 (100%)	100%
RM 2	63.4	5/5 (100%)	5/5 (100%)	100%
RM 3	39.6	5/5 (100%)	5/5 (100%)	100%

Supplementary Table 6: Cloning efficiency of ChrI segments 1 through 3 from BY, RM, and *S.paradoxus*. Column 2 ('Size') lists the size of each target segment from all 3 different strains. Column 2 ('Upstream junction check') lists the number of positive PCR reactions from the upstream junction of a target segment and the capture vector. Column 3 ('Downstream junction check') lists the number of positive PCR reactions from the downstream junction of a target segment and the capture vector. Column 4 ('Cloning efficiency') lists the overall efficiency of segment cloning based on the positive rate of PCR reaction on both junctions up and downstream.

Strain	Segment 1	Segment 2	Segment 3	Assembly Efficiency	Native ChrI elimination efficiency
IEY394	*S.par*	*S.par*	*S.par*	4/5 (75%)	4/4 (100%)
IEY395	*S.par*	RM	*S.par*	3/3 (100%)	3/3 (100%)
IEY396	*S.par*	*S.par*	RM	3/3 (100%)	2/3 (66%)
IEY397	*S.par*	RM	RM	1/3 (33%)	1/1 (100%)
IEY398	RM	RM	RM	2/3 (66%)	2/2 (100%)
IEY399	RM	*S.par*	RM	2/3 (66%)	2/2 (100%)
IEY400	RM	RM	*S.par*	1/3 (33%)	1/1 (100%)
IEY401	RM	*S.par*	*S.par*	3/3 (100%)	2/3 (66%)
IEY402	BY	BY	BY	2/3 (66%)	1/2 (50%)
IEY403	BY	BY	RM	1/5 (20%)	1/1 (100%)
IEY404	BY	BY	*S.par*	2/5 (40%)	1/2 (50%)
IEY405	BY	RM	BY	2/3 (66%)	2/2 (100%)
IEY406	BY	RM	RM	1/3 (33%)	1/1 (100%)
IEY407	BY	RM	*S.par*	2/3 (66%)	2/2 (100%)
IEY408	BY	*S.par*	BY	2/3 (66%)	2/2 (100%)
IEY409	BY	*S.par*	RM	3/3 (100%)	3/3 (100%)
IEY410	BY	*S.par*	*S.par*	2/3 (66%)	2/2 (100%)
IEY411	*S.par*	BY	*S.par*	3/3 (100%)	2/3 (66%)
IEY412	*S.par*	BY	RM	2/3 (66%)	2/2 (100%)
IEY413	*S.par*	BY	BY	2/3 (66%)	1/2 (50%)
IEY414	*S.par*	*S.par*	BY	2/3 (66%)	2/2 (100%)
IEY415	*S.par*	RM	BY	2/3 (66%)	2/2 (100%)

IEY416	RM	BY	BY	1/3 (33%)	1/1 (100%)
IEY417	RM	BY	RM	2/3(66%)	2/2(100%)
IEY418	RM	BY	*S.par*	2/3 (66%)	1/2 (50%)
IEY419	RM	RM	BY	1/3 (33%)	1/1 (100%)
IEY420	RM	*S.par*	BY	2/3 (66%)	2/2 (100%)

Supplementary Table 7: Efficiency of assembly of synthetic chimera ChrI in BY4742 and elimination of its native ChrI. Column 1 ('Strain') lists the ID of all the strains containing chimeric versions of ChrI. Column 2-4 ('Segment 1', 'Segment 2' and 'Segment 3') lists the strain specific segment associated to each chimera ChrI in distinct chimera strains. Column 5 ('Assembly efficiency') lists the efficiency of assembling the synthetic ChrI for each chimera cell based on PCR checking of 5 junctions across the assembled ChrI. Column 6 ('Native ChrI elimination efficiency') lists the efficiency of elimination of native BY ChrI based on checking of regions in the native ChrI.

Strain	ChrI genotype	depth coverage	std	number of mutations	mutation location (reference genome)
Saccharomyces cerevisiae BY4742	Reference	139.0	33.1	NA	NA
Saccharomyces cerevisiae RM11-1a	Reference	147.8	32.3	NA	NA
Saccharomyces paradoxus CBS5829	Reference	86.5	21.7	NA	NA
Aneuploid_BY_ *Spar* ChrI	BY ChrI + synthetic *Spar* ChrI	103.5	27.9	1	A:G_123929
IEY394	PAR1_PAR2_PAR3	85.3	17.8	1	A:G_123929
IEY395	PAR1_RM2_PAR3	340.8	190.1	4	T:G_61428;A:G_123612;A:G_163088;A:G_163090
IEY396	PAR1_PAR2_RM3	340.8	190.1	2	A:G_123929;G:A_163558
IEY397	PAR1_RM2_RM3	128.2	50.7	2	A:G_123612;G:A_163241
IEY398	RM1_RM2_RM3	72.3	41.2	3	A:G_123496;G:A_163125;G:A_163154
IEY399	RM1_PAR2_RM3	112.3	47.4	3	A:G_123813;G:A_163442;G:A_163471
IEY400	RM1_RM2_PAR3	36.8	15.8	2	G:A_121884;A:G_123496
IEY402	BY1_BY2_BY3	156.8	13.2	3	T:G_10300;A:T_19634;A:G_123477
IEY403	BY1_BY2_RM3	57.4	13.1	3	T:G_10300;A:G_123477;G:A_163106
IEY404	BY1_BY2_PAR3	122.8	14.8	1	T:G_10300
IEY405	BY1_RM2_BY3	17.6	4.0	NA	NA
IEY407	BY1_RM2_PAR3	121.6	11.8	1	T:G_10300
IEY408	BY1_PAR2_BY3	121.9	15.9	3	A:G_123480;G:A_163109;G:A_163138
IEY409	BY1_PAR2_RM3	53.6	7.6	3	T:G_10300;A:G_123810;G:A_163439

IEY410	BY1_PAR2_PAR3	26.2	4.2	2	T:G_10300;A:G_123810
IEY411	PAR1_BY2_PAR3	84.3	9.0	1	A:G_123596
IEY412	PAR1_BY2_RM3	61.4	7.4	3	A:G_123596;G:A_163225;G:A_163254
IEY413	PAR1_BY2_BY3	91.9	9.8	2	A:G_123596;G:A_168734
IEY414	PAR1_PAR2_BY3	32.4	4.9	3	C:T_120611;A:G_123929;G:A_169067
IEY415	PAR1_RM2_BY3	114.0	82.9	2	A:G_123612;G:A_168750
IEY416	RM1_BY2_BY3	14.4	4.1	NA	NA
IEY417	RM1_BY2_RM3	120.8	9.9	3	A:G_123480;G:A_163109;G:A163138
IEY418	RM1_BY2_PAR3	36.8	7.3	0	
IEY420	RM1_PAR2_BY3	20.8	3.6	NA	NA
IEY421	Seg1_Seg3_Seg2	87.5	10.8	1	T:G_17763
IEY422	Seg2_Seg1_Seg3	74.6	19.1	3	T:G_1828;A:G_130844;G:A_175984
IEY423	Seg2_Seg3_Seg1	22.8	4.6	NA	NA
IEY425	Seg3_Seg1_Seg2	75.1	10.0	0	
IEY426	Multiple Deletion ChrI	39.1	6.8	0	

Supplementary Table 8: Depth coverage and mutation numbers of all synthetic strains sequenced using ONT data. Column 1 ('Strain') lists the ID of all the sequenced strains including donor strains. Column 2 (‘ChrI genotype’) lists the ChrI genotype of each sequenced strain. ChrI genotypes from donor strains are marked as ‘Reference’. Column 3 ('Depth coverage') lists the average depth coverage of ChrI on each sequenced strain. Column 4 ('std') lists the standard deviation associated with depth coverage on ChrI for each sequenced strain. Column 5 (‘number of mutations’) lists the total number of mutations present on each synthesized ChrI when compared to its reference ChrI. Column 6 ('mutation location (reference genome') lists the position of each mutation according to the reference ChrI and also the nucleotide change observed on each position. Synthetic chromosomes whose coverage was below 21x were excluded from nucleotide variant calling.

Strain	Segment 1	Segment 2	Segment 3	Doubling time at 30ºC (min)	Doubling time at 35ºC (min)
IEY394	*S.par*	*S.par*	*S.par*	111.44	165.08
IEY394	*S.par*	*S.par*	*S.par*	134.50	149.37
IEY394	*S.par*	*S.par*	*S.par*	108.43	140.56
IEY394	*S.par*	*S.par*	*S.par*	171.57	141.03
IEY394	*S.par*	*S.par*	*S.par*	107.16	146.18
IEY394	*S.par*	*S.par*	*S.par*	108.88	138.49
IEY394	*S.par*	*S.par*	*S.par*	96.88	137.65
IEY394	*S.par*	*S.par*	*S.par*	90.21	132.13
IEY394	*S.par*	*S.par*	*S.par*	90.86	141.55
IEY394	*S.par*	*S.par*	*S.par*	109.07	140.61
IEY394	*S.par*	*S.par*	*S.par*	112.41	133.81
IEY394	*S.par*	*S.par*	*S.par*	113.71	131.89
IEY395	*S.par*	RM	*S.par*	115.39	129.06
IEY395	*S.par*	RM	*S.par*	117.54	114.86
IEY395	*S.par*	RM	*S.par*	114.86	114.20
IEY395	*S.par*	RM	*S.par*	153.70	113.91
IEY395	*S.par*	RM	*S.par*	106.71	116.01
IEY395	*S.par*	RM	*S.par*	114.86	116.22
IEY395	*S.par*	RM	*S.par*	94.59	109.02
IEY395	*S.par*	RM	*S.par*	92.16	132.85
IEY395	*S.par*	RM	*S.par*	93.07	113.63
IEY395	*S.par*	RM	*S.par*	107.01	115.22
IEY395	*S.par*	RM	*S.par*	152.05	112.60
IEY395	*S.par*	RM	*S.par*	107.78	111.27
IEY396	*S.par*	*S.par*	RM	141.48	100.33

IEY396	*S.par*	*S.par*	RM	101.89	105.56
IEY396	*S.par*	*S.par*	RM	97.58	97.22
IEY396	*S.par*	*S.par*	RM	128.94	101.20
IEY396	*S.par*	*S.par*	RM	101.69	100.53
IEY396	*S.par*	*S.par*	RM	97.24	93.82
IEY396	*S.par*	*S.par*	RM	84.27	109.88
IEY396	*S.par*	*S.par*	RM	78.61	110.05
IEY396	*S.par*	*S.par*	RM	78.55	113.75
IEY396	*S.par*	*S.par*	RM	107.67	99.85
IEY396	*S.par*	*S.par*	RM	108.14	96.66
IEY396	*S.par*	*S.par*	RM	110.76	94.51
IEY397	*S.par*	RM	RM	163.53	92.15
IEY397	*S.par*	RM	RM	127.22	109.96
IEY397	*S.par*	RM	RM	106.07	96.31
IEY397	*S.par*	RM	RM	100.38	97.90
IEY397	*S.par*	RM	RM	106.61	99.11
IEY397	*S.par*	RM	RM	101.13	102.54
IEY397	*S.par*	RM	RM	82.23	91.80
IEY397	*S.par*	RM	RM	80.79	94.46
IEY397	*S.par*	RM	RM	78.43	98.25
IEY397	*S.par*	RM	RM	89.65	95.67
IEY397	*S.par*	RM	RM	103.43	106.20
IEY397	*S.par*	RM	RM	107.11	96.06
IEY398	RM	RM	RM	193.21	88.70
IEY398	RM	RM	RM	106.67	110.05
IEY398	RM	RM	RM	97.48	91.66
IEY398	RM	RM	RM	120.40	93.87
IEY398	RM	RM	RM	108.35	99.16

IEY398	RM	RM	RM	96.34	96.00
IEY398	RM	RM	RM	79.69	116.07
IEY398	RM	RM	RM	86.30	125.08
IEY398	RM	RM	RM	78.76	114.31
IEY398	RM	RM	RM	107.01	94.95
IEY398	RM	RM	RM	131.56	104.81
IEY398	RM	RM	RM	109.91	90.97
IEY399	RM	*S.par*	RM	114.74	110.19
IEY399	RM	*S.par*	RM	116.44	112.38
IEY399	RM	*S.par*	RM	109.13	107.33
IEY399	RM	*S.par*	RM	115.76	113.84
IEY399	RM	*S.par*	RM	102.88	111.43
IEY399	RM	*S.par*	RM	107.20	113.95
IEY399	RM	*S.par*	RM	94.40	120.90
IEY399	RM	*S.par*	RM	92.74	120.19
IEY399	RM	*S.par*	RM	86.28	119.08
IEY399	RM	*S.par*	RM	112.55	107.46
IEY399	RM	*S.par*	RM	123.36	106.58
IEY399	RM	*S.par*	RM	119.01	103.29
IEY400	RM	RM	*S.par*	103.70	91.70
IEY400	RM	RM	*S.par*	100.39	89.97
IEY400	RM	RM	*S.par*	103.55	87.86
IEY400	RM	RM	*S.par*	101.79	99.19
IEY400	RM	RM	*S.par*	97.95	97.10
IEY400	RM	RM	*S.par*	108.34	97.16
IEY400	RM	RM	*S.par*	84.52	90.03
IEY400	RM	RM	*S.par*	81.97	92.55
IEY400	RM	RM	*S.par*	81.09	95.57

IEY400	RM	RM	*S.par*	97.74	94.86
IEY400	RM	RM	*S.par*	94.87	93.30
IEY400	RM	RM	*S.par*	95.65	96.17
IEY401	RM	*S.par*	*S.par*	96.34	105.44
IEY401	RM	*S.par*	*S.par*	111.43	102.98
IEY401	RM	*S.par*	*S.par*	104.02	105.55
IEY401	RM	*S.par*	*S.par*	122.67	105.08
IEY401	RM	*S.par*	*S.par*	123.57	113.44
IEY401	RM	*S.par*	*S.par*	99.25	103.67
IEY401	RM	*S.par*	*S.par*	83.59	153.78
IEY401	RM	*S.par*	*S.par*	79.26	131.62
IEY401	RM	*S.par*	*S.par*	82.72	144.71
IEY401	RM	*S.par*	*S.par*	105.90	126.25
IEY401	RM	*S.par*	*S.par*	108.08	114.88
IEY401	RM	*S.par*	*S.par*	110.53	121.76
IEY402	BY	BY	BY	100.73	93.58
IEY402	BY	BY	BY	134.91	91.15
IEY402	BY	BY	BY	98.83	91.61
IEY402	BY	BY	BY	98.59	99.59
IEY402	BY	BY	BY	107.57	99.90
IEY402	BY	BY	BY	97.05	99.31
IEY402	BY	BY	BY	79.31	94.74
IEY402	BY	BY	BY	79.51	99.55
IEY402	BY	BY	BY	82.44	96.03
IEY402	BY	BY	BY	97.04	99.24
IEY402	BY	BY	BY	100.13	96.04
IEY402	BY	BY	BY	94.55	94.92
IEY403	BY	BY	RM	104.38	95.21

IEY403	BY	BY	RM	98.45	89.49
IEY403	BY	BY	RM	98.11	93.03
IEY403	BY	BY	RM	97.57	98.20
IEY403	BY	BY	RM	95.08	97.17
IEY403	BY	BY	RM	96.55	97.95
IEY403	BY	BY	RM	80.10	98.51
IEY403	BY	BY	RM	78.02	91.58
IEY403	BY	BY	RM	79.91	100.46
IEY403	BY	BY	RM	98.98	95.35
IEY403	BY	BY	RM	96.09	97.72
IEY403	BY	BY	RM	92.36	95.99
IEY404	BY	BY	*S.par*	100.57	89.86
IEY404	BY	BY	*S.par*	95.16	92.63
IEY404	BY	BY	*S.par*	105.89	92.34
IEY404	BY	BY	*S.par*	95.67	99.15
IEY404	BY	BY	*S.par*	84.81	93.95
IEY404	BY	BY	*S.par*	141.96	97.09
IEY404	BY	BY	*S.par*	77.73	96.28
IEY404	BY	BY	*S.par*	74.52	92.79
IEY404	BY	BY	*S.par*	83.45	89.94
IEY404	BY	BY	*S.par*	96.24	111.71
IEY404	BY	BY	*S.par*	98.27	111.13
IEY404	BY	BY	*S.par*	96.87	113.70
IEY405	BY	RM	BY	150.58	89.89
IEY405	BY	RM	BY	97.66	93.34
IEY405	BY	RM	BY	94.45	88.26
IEY405	BY	RM	BY	135.82	95.63
IEY405	BY	RM	BY	95.62	93.16

IEY405	BY	RM	BY	99.28	91.42
IEY405	BY	RM	BY	83.12	100.11
IEY405	BY	RM	BY	73.78	96.78
IEY405	BY	RM	BY	74.79	96.21
IEY405	BY	RM	BY	99.23	92.95
IEY405	BY	RM	BY	98.31	99.88
IEY405	BY	RM	BY	97.62	96.07
IEY406	BY	RM	RM	98.64	90.42
IEY406	BY	RM	RM	99.96	97.39
IEY406	BY	RM	RM	103.43	92.24
IEY406	BY	RM	RM	97.69	98.47
IEY406	BY	RM	RM	98.96	98.65
IEY406	BY	RM	RM	107.56	106.07
IEY406	BY	RM	RM	81.10	98.12
IEY406	BY	RM	RM	80.45	95.23
IEY406	BY	RM	RM	86.00	99.96
IEY406	BY	RM	RM	97.24	91.73
IEY406	BY	RM	RM	98.77	95.17
IEY406	BY	RM	RM	107.68	96.90
IEY407	BY	RM	*S.par*	103.71	114.27
IEY407	BY	RM	*S.par*	108.85	111.31
IEY407	BY	RM	*S.par*	107.67	107.95
IEY407	BY	RM	*S.par*	103.68	111.91
IEY407	BY	RM	*S.par*	109.95	111.00
IEY407	BY	RM	*S.par*	103.46	114.86
IEY407	BY	RM	*S.par*	80.30	150.23
IEY407	BY	RM	*S.par*	85.17	159.99
IEY407	BY	RM	*S.par*	86.51	148.75

IEY407	BY	RM	*S.par*	150.37	114.09
IEY407	BY	RM	*S.par*	155.89	110.30
IEY407	BY	RM	*S.par*	151.36	115.41
IEY408	BY	*S.par*	BY	90.42	85.75
IEY408	BY	*S.par*	BY	94.23	84.32
IEY408	BY	*S.par*	BY	103.81	83.90
IEY408	BY	*S.par*	BY	90.35	86.98
IEY408	BY	*S.par*	BY	92.88	89.61
IEY408	BY	*S.par*	BY	91.66	87.60
IEY408	BY	*S.par*	BY	73.21	86.86
IEY408	BY	*S.par*	BY	76.60	86.56
IEY408	BY	*S.par*	BY	74.02	82.62
IEY408	BY	*S.par*	BY	90.13	86.21
IEY408	BY	*S.par*	BY	90.40	87.49
IEY408	BY	*S.par*	BY	90.65	88.90
IEY409	BY	*S.par*	RM	95.71	80.46
IEY409	BY	*S.par*	RM	96.66	84.94
IEY409	BY	*S.par*	RM	97.24	84.59
IEY409	BY	*S.par*	RM	111.19	89.25
IEY409	BY	*S.par*	RM	93.64	88.38
IEY409	BY	*S.par*	RM	94.55	89.38
IEY409	BY	*S.par*	RM	74.41	85.89
IEY409	BY	*S.par*	RM	74.02	86.26
IEY409	BY	*S.par*	RM	76.05	89.58
IEY409	BY	*S.par*	RM	93.41	88.87
IEY409	BY	*S.par*	RM	90.63	87.04
IEY409	BY	*S.par*	RM	96.19	89.56
IEY410	BY	*S.par*	*S.par*	99.23	92.58

IEY410	BY	*S.par*	*S.par*	99.32	93.79
IEY410	BY	*S.par*	*S.par*	97.82	91.94
IEY410	BY	*S.par*	*S.par*	96.19	97.97
IEY410	BY	*S.par*	*S.par*	106.00	97.81
IEY410	BY	*S.par*	*S.par*	98.09	94.37
IEY410	BY	*S.par*	*S.par*	77.93	96.06
IEY410	BY	*S.par*	*S.par*	78.50	96.48
IEY410	BY	*S.par*	*S.par*	81.09	98.55
IEY410	BY	*S.par*	*S.par*	95.59	94.81
IEY410	BY	*S.par*	*S.par*	91.86	94.21
IEY410	BY	*S.par*	*S.par*	91.11	95.25
IEY411	*S.par*	BY	*S.par*	139.00	106.99
IEY411	*S.par*	BY	*S.par*	105.68	109.25
IEY411	*S.par*	BY	*S.par*	103.82	108.37
IEY411	*S.par*	BY	*S.par*	103.24	119.22
IEY411	*S.par*	BY	*S.par*	103.22	109.96
IEY411	*S.par*	BY	*S.par*	103.19	109.30
IEY411	*S.par*	BY	*S.par*	83.73	113.16
IEY411	*S.par*	BY	*S.par*	89.59	109.83
IEY411	*S.par*	BY	*S.par*	87.08	97.96
IEY411	*S.par*	BY	*S.par*	103.48	104.34
IEY411	*S.par*	BY	*S.par*	96.79	107.79
IEY411	*S.par*	BY	*S.par*	101.25	108.55
IEY412	*S.par*	BY	RM	95.21	90.40
IEY412	*S.par*	BY	RM	93.95	91.75
IEY412	*S.par*	BY	RM	105.71	87.36
IEY412	*S.par*	BY	RM	92.19	96.81
IEY412	*S.par*	BY	RM	94.98	95.19

IEY412	*S.par*	BY	RM	112.75	94.28
IEY412	*S.par*	BY	RM	74.02	90.03
IEY412	*S.par*	BY	RM	74.32	91.88
IEY412	*S.par*	BY	RM	76.20	85.32
IEY412	*S.par*	BY	RM	87.36	90.15
IEY412	*S.par*	BY	RM	90.35	89.91
IEY412	*S.par*	BY	RM	94.10	92.06
IEY413	*S.par*	BY	BY	125.41	92.98
IEY413	*S.par*	BY	BY	102.03	91.16
IEY413	*S.par*	BY	BY	97.33	98.29
IEY413	*S.par*	BY	BY	95.77	97.89
IEY413	*S.par*	BY	BY	113.60	100.17
IEY413	*S.par*	BY	BY	98.10	99.37
IEY413	*S.par*	BY	BY	78.05	100.01
IEY413	*S.par*	BY	BY	77.70	92.95
IEY413	*S.par*	BY	BY	80.50	97.17
IEY413	*S.par*	BY	BY	108.41	93.21
IEY413	*S.par*	BY	BY	91.55	91.25
IEY413	*S.par*	BY	BY	98.40	93.03
IEY414	*S.par*	*S.par*	BY	109.75	115.35
IEY414	*S.par*	*S.par*	BY	107.50	116.79
IEY414	*S.par*	*S.par*	BY	181.90	107.08
IEY414	*S.par*	*S.par*	BY	107.29	115.33
IEY414	*S.par*	*S.par*	BY	108.19	113.85
IEY414	*S.par*	*S.par*	BY	141.57	110.15
IEY414	*S.par*	*S.par*	BY	83.85	125.41
IEY414	*S.par*	*S.par*	BY	85.84	110.89
IEY414	*S.par*	*S.par*	BY	91.67	114.56

IEY414	*S.par*	*S.par*	BY	102.61	96.99
IEY414	*S.par*	*S.par*	BY	105.53	99.04
IEY414	*S.par*	*S.par*	BY	137.91	98.52
IEY415	*S.par*	RM	BY	100.30	96.59
IEY415	*S.par*	RM	BY	101.86	94.89
IEY415	*S.par*	RM	BY	110.77	91.46
IEY415	*S.par*	RM	BY	99.98	98.98
IEY415	*S.par*	RM	BY	99.53	99.53
IEY415	*S.par*	RM	BY	110.56	98.24
IEY415	*S.par*	RM	BY	79.62	93.86
IEY415	*S.par*	RM	BY	83.55	98.18
IEY415	*S.par*	RM	BY	83.43	93.47
IEY415	*S.par*	RM	BY	93.84	98.54
IEY415	*S.par*	RM	BY	100.18	98.69
IEY415	*S.par*	RM	BY	96.10	98.00
IEY416	RM	BY	BY	102.25	91.42
IEY416	RM	BY	BY	99.58	88.05
IEY416	RM	BY	BY	101.93	88.97
IEY416	RM	BY	BY	99.51	96.98
IEY416	RM	BY	BY	96.00	94.96
IEY416	RM	BY	BY	99.08	96.30
IEY416	RM	BY	BY	80.06	93.04
IEY416	RM	BY	BY	81.26	88.82
IEY416	RM	BY	BY	83.34	93.87
IEY416	RM	BY	BY	93.37	91.22
IEY416	RM	BY	BY	92.10	94.51
IEY416	RM	BY	BY	95.75	88.39
IEY417	RM	BY	RM	100.16	91.48

IEY417	RM	BY	RM	97.36	93.09
IEY417	RM	BY	RM	99.78	91.39
IEY417	RM	BY	RM	98.90	98.07
IEY417	RM	BY	RM	99.47	97.66
IEY417	RM	BY	RM	123.89	94.00
IEY417	RM	BY	RM	80.23	96.26
IEY417	RM	BY	RM	79.48	95.25
IEY417	RM	BY	RM	78.48	98.38
IEY417	RM	BY	RM	95.93	94.61
IEY417	RM	BY	RM	93.44	93.98
IEY417	RM	BY	RM	97.87	95.14
IEY418	RM	BY	*S.par*	103.16	110.37
IEY418	RM	BY	*S.par*	99.66	106.30
IEY418	RM	BY	*S.par*	100.88	104.78
IEY418	RM	BY	*S.par*	99.60	118.36
IEY418	RM	BY	*S.par*	100.01	112.71
IEY418	RM	BY	*S.par*	123.84	116.38
IEY418	RM	BY	*S.par*	81.33	100.52
IEY418	RM	BY	*S.par*	81.48	103.17
IEY418	RM	BY	*S.par*	77.98	106.82
IEY418	RM	BY	*S.par*	97.73	102.41
IEY418	RM	BY	*S.par*	93.84	106.34
IEY418	RM	BY	*S.par*	99.22	110.66
IEY419	RM	RM	BY	98.97	93.49
IEY419	RM	RM	BY	102.52	88.93
IEY419	RM	RM	BY	96.82	95.47
IEY419	RM	RM	BY	101.67	97.57
IEY419	RM	RM	BY	105.74	98.14

IEY419	RM	RM	BY	98.89	98.90
IEY419	RM	RM	BY	83.19	93.09
IEY419	RM	RM	BY	82.34	94.19
IEY419	RM	RM	BY	81.41	97.76
IEY419	RM	RM	BY	92.21	94.96
IEY419	RM	RM	BY	91.60	96.19
IEY419	RM	RM	BY	93.95	99.79
IEY420	RM	*S.par*	BY	114.00	107.09
IEY420	RM	*S.par*	BY	93.23	112.72
IEY420	RM	*S.par*	BY	105.20	108.76
IEY420	RM	*S.par*	BY	95.00	118.66
IEY420	RM	*S.par*	BY	94.19	116.45
IEY420	RM	*S.par*	BY	92.51	116.09
IEY420	RM	*S.par*	BY	77.23	101.59
IEY420	RM	*S.par*	BY	73.77	109.68
IEY420	RM	*S.par*	BY	73.40	107.20
IEY420	RM	*S.par*	BY	103.73	104.83
IEY420	RM	*S.par*	BY	111.66	111.03
IEY420	RM	*S.par*	BY	108.06	112.43

Supplementary Table 9: Phenotypic assay of all BY ChrI chimera strains growing on rich media at distinct temperature, 30 and 35ºC. Column I ('Strain') lists IDs of each chimera strain. Column 2 through 4 ('Segment1', 'Segment2' and 'Segment3') lists each strain-specific segment to its respective chimera strain. Column 5 ('Doubling time at 30ºC (min)') lists the doubling time of each chimera strain growing on rich media at 30ºC. Column 5 ('Doubling time at 35ºC (min)') lists the doubling time of each chimera strain growing on rich media at 35ºC.

	Df	Sum Sq	Mean Sq	F value	Pr(>F)	PVE(%)
**Segment 1**	2	1.539	0.7694	52.452	< 2e-16 ***	8.9
**Segment 2**	2	1.085	0.5425	36.985	5.18e-15 ***	6.3
**Segment 3**	2	3.784	1.8922	129.003	< 2e-16 ***	22
**Segment1;Segment2**	4	3.594	0.8985	61.256	< 2e-16 ***	20
**Segment1;Segment3**	4	1.253	0.3132	21.355	1.99e-15 ***	7.3
**Segment2;Segment3**	4	0.314	0.0786	5.365	0.000358 ***	1.8
**Segment1;Segment2;Segment3**	7	1.394	0.1992	13.579	3.76e-15 ***	8.1
**Residuals**	286	4.195	0.0147			

Supplementary Table 10: Full factorial ANOVA table for growth of chimera ChrI cells at 35ºC. PVE (phenotypic variance explained). Interaction terms are denoted by ‘ ; ’.

Strain	Position 1	Position 2	Position 3	Assembly efficiency	Native ChrI elimination efficiency
IEY421	Seg1	Seg3	Seg2	1/3 (33%)	1/1(100%)
IEY422	Seg2	Seg1	Seg3	3/3 (100%)	2/3 (66%)
IEY423	Seg2	Seg3	Seg1	3/3 (100%)	3/3(100%)
IEY424	Seg3	Seg2	Seg1	3/3 (100%)	3/3(100%)
IEY425	Seg3	Seg1	Seg2	2/3 (66%)	2/2 (100%)

Supplementary Table 11: Efficiency of assembly of synthetic restructured ChrI in BY and elimination of its native ChrI. Column 1 ('Strain') lists the ID of all the strains containing restructured versions of ChrI. Column 2-4 ('Position1','Position2' and 'Position3') lists the order of assembled segments for each restructured version of ChrI. Column 5 ('Assembly efficiency') lists the efficiency of assembling the restructured ChrI for each strain cell based on PCR checking of 5 junctions across the assembled ChrI. Column 6 ('Native ChrI elimination efficiency') lists the efficiency of elimination of native BY ChrI based on PCR checking of segments covering the native ChrI. A total of 12 replicas per strain were phenotyped on both conditions.

Strain	Position 1	Position 2	Position 3	Doubling time
IEY402	Seg1	Seg2	Seg3	96.62
IEY402	Seg1	Seg2	Seg3	93.60
IEY402	Seg1	Seg2	Seg3	94.60
IEY402	Seg1	Seg2	Seg3	90.30
IEY402	Seg1	Seg2	Seg3	92.72
IEY402	Seg1	Seg2	Seg3	92.21
IEY402	Seg1	Seg2	Seg3	91.19
IEY402	Seg1	Seg2	Seg3	92.23
IEY402	Seg1	Seg2	Seg3	87.94
IEY402	Seg1	Seg2	Seg3	92.46
IEY402	Seg1	Seg2	Seg3	93.59
IEY402	Seg1	Seg2	Seg3	87.00
IEY421	Seg1	Seg3	Seg2	159.29
IEY421	Seg1	Seg3	Seg2	154.82
IEY421	Seg1	Seg3	Seg2	154.62
IEY421	Seg1	Seg3	Seg2	158.20
IEY421	Seg1	Seg3	Seg2	152.05
IEY421	Seg1	Seg3	Seg2	154.72
IEY421	Seg1	Seg3	Seg2	172.11
IEY421	Seg1	Seg3	Seg2	159.60
IEY421	Seg1	Seg3	Seg2	154.00
IEY421	Seg1	Seg3	Seg2	161.71
IEY421	Seg1	Seg3	Seg2	155.03
IEY421	Seg1	Seg3	Seg2	152.42

IEY422	Seg2	Seg1	Seg3	94.50
IEY422	Seg2	Seg1	Seg3	91.72
IEY422	Seg2	Seg1	Seg3	92.10
IEY422	Seg2	Seg1	Seg3	100.66
IEY422	Seg2	Seg1	Seg3	94.66
IEY422	Seg2	Seg1	Seg3	92.03
IEY422	Seg2	Seg1	Seg3	95.01
IEY422	Seg2	Seg1	Seg3	89.22
IEY422	Seg2	Seg1	Seg3	93.12
IEY422	Seg2	Seg1	Seg3	96.33
IEY422	Seg2	Seg1	Seg3	91.71
IEY422	Seg2	Seg1	Seg3	92.06
IEY423	Seg2	Seg3	Seg1	85.52
IEY423	Seg2	Seg3	Seg1	86.26
IEY423	Seg2	Seg3	Seg1	87.82
IEY423	Seg2	Seg3	Seg1	83.06
IEY423	Seg2	Seg3	Seg1	82.81
IEY423	Seg2	Seg3	Seg1	86.28
IEY423	Seg2	Seg3	Seg1	85.52
IEY423	Seg2	Seg3	Seg1	84.91
IEY423	Seg2	Seg3	Seg1	87.38
IEY423	Seg2	Seg3	Seg1	85.61
IEY423	Seg2	Seg3	Seg1	89.01
IEY423	Seg2	Seg3	Seg1	87.38
IEY424	Seg3	Seg2	Seg1	89.37

IEY424	Seg3	Seg2	Seg1	88.97
IEY424	Seg3	Seg2	Seg1	92.54
IEY424	Seg3	Seg2	Seg1	89.60
IEY424	Seg3	Seg2	Seg1	90.31
IEY424	Seg3	Seg2	Seg1	94.11
IEY424	Seg3	Seg2	Seg1	90.14
IEY424	Seg3	Seg2	Seg1	95.18
IEY424	Seg3	Seg2	Seg1	93.23
IEY424	Seg3	Seg2	Seg1	90.53
IEY424	Seg3	Seg2	Seg1	95.33
IEY424	Seg3	Seg2	Seg1	93.61
IEY425	Seg3	Seg1	Seg2	113.38
IEY425	Seg3	Seg1	Seg2	111.67
IEY425	Seg3	Seg1	Seg2	110.92
IEY425	Seg3	Seg1	Seg2	103.61
IEY425	Seg3	Seg1	Seg2	104.70
IEY425	Seg3	Seg1	Seg2	109.69
IEY425	Seg3	Seg1	Seg2	104.39
IEY425	Seg3	Seg1	Seg2	105.12
IEY425	Seg3	Seg1	Seg2	111.11
IEY425	Seg3	Seg1	Seg2	107.13
IEY425	Seg3	Seg1	Seg2	107.45
IEY425	Seg3	Seg1	Seg2	110.82

Supplementary Table 12: Phenotypic assay of strains containing the restructured versions of BY ChrI. Column 1 ('Strain') lists IDs of each strain carrying a restructured version of ChrI. Column 2-4 ('Position 1','Position 2' and 'Position 3') lists the order of assembled segments for each restructured version

of ChrI. Column 5 ('Doubling time at 30ºC (min)') lists the doubling time of each restructured ChrI strain growing on rich media at 30ºC. A total of nine replicates per strain were phenotyped.

Gene	Strain	average Ct	ΔCt
NUP60	IEY402	15.44	0.18
NUP60	IEY402	15.68	-0.06
NUP60	IEY402	15.66	-0.04
NUP60	IEY402	15.71	-0.09
NUP60	IEY421	15.43	0.19
NUP60	IEY421	15.18	0.45
NUP60	IEY421	15.48	0.14
NUP60	IEY421	15.66	-0.03
NUP60	IEY425	15.66	-0.04
NUP60	IEY425	15.67	-0.04
NUP60	IEY425	15.37	0.25
NUP60	IEY425	15.60	0.02
MYO4	IEY402	15.52	0.16
MYO4	IEY402	15.51	0.17
MYO4	IEY402	15.97	-0.29
MYO4	IEY402	15.71	-0.03
MYO4	IEY421	15.68	0.00
MYO4	IEY421	15.40	0.28
MYO4	IEY421	15.70	-0.02
MYO4	IEY421	15.76	-0.08
MYO4	IEY425	15.54	0.14
MYO4	IEY425	15.38	0.29
MYO4	IEY425	15.18	0.50
MYO4	IEY425	15.29	0.38
SNC1	IEY402	16.45	-0.13
SNC1	IEY402	16.06	0.26
SNC1	IEY402	16.43	-0.11
SNC1	IEY402	16.36	-0.03
SNC1	IEY421	15.10	1.22
SNC1	IEY421	16.31	0.01
SNC1	IEY421	15.52	0.80
SNC1	IEY421	15.38	0.95
SNC1	IEY425	15.54	0.78
SNC1	IEY425	16.38	-0.06
SNC1	IEY425	15.57	0.76
SNC1	IEY425	15.57	0.76

ACT1	IEY402	15.64	0.29
ACT1	IEY402	16.21	-0.28
ACT1	IEY402	15.94	-0.01
ACT1	IEY402	15.95	-0.02
ACT1	IEY421	16.03	-0.10
ACT1	IEY421	16.13	-0.20
ACT1	IEY421	16.76	-0.83
ACT1	IEY421	16.41	-0.47
ACT1	IEY425	15.95	-0.02
ACT1	IEY425	15.64	0.29
ACT1	IEY425	15.66	0.27
ACT1	IEY425	15.62	0.32

Supplementary Table 13: Ct and ΔCt values of genes *MYO4*, *SNC1*, *NUP60*, and *ACT1* for ChrI restructured strains 1-3-2 (IEY421), 3-1-2 (IEY425) and 1-2-3 (IEY402) generated during RT-qPCR assay. Column 1 ('Gene') lists IDs of each analyzed gene. Column 2 (‘Strain’) lists IDs of each analyzed strain. Column 3 ('Ct') lists Ct values obtained for each gene per strain in four biological replicas. Ct values were automatically calculated by the software AriaMx HRM qPCR. Column 5 ('**Δ**Ct') lists the **Δ**Ct of each analyzed gene calculated according to the Pfaffl method.

Gene	Strain	Position 1	Position 2	Position 3	Gene expression ratio
NUP60	IEY402	seg1	seg2	seg3	0.926743422
NUP60	IEY402	seg1	seg2	seg3	1.172498027
NUP60	IEY402	seg1	seg2	seg3	0.974790435
NUP60	IEY402	seg1	seg2	seg3	0.944098067
NUP60	IEY421	seg1	seg3	seg2	1.249838494
NUP60	IEY421	seg1	seg3	seg2	1.646716829
NUP60	IEY421	seg1	seg3	seg2	2.072330194
NUP60	IEY421	seg1	seg3	seg2	1.385932684
NUP60	IEY425	seg3	seg1	seg2	0.982096319
NUP60	IEY425	seg3	seg1	seg2	0.776819492
NUP60	IEY425	seg3	seg1	seg2	0.994064922
NUP60	IEY425	seg3	seg1	seg2	0.799776236
MYO4	IEY402	seg1	seg2	seg3	1.013267885
MYO4	IEY402	seg1	seg2	seg3	0.998496093
MYO4	IEY402	seg1	seg2	seg3	0.895846458
MYO4	IEY402	seg1	seg2	seg3	1.103305469
MYO4	IEY421	seg1	seg3	seg2	1.198354725
MYO4	IEY421	seg1	seg3	seg2	1.609976715
MYO4	IEY421	seg1	seg3	seg2	2.035215749
MYO4	IEY421	seg1	seg3	seg2	1.493992798
MYO4	IEY425	seg3	seg1	seg2	1.257623688
MYO4	IEY425	seg3	seg1	seg2	1.127583306

MYO4	IEY425	seg3	seg1	seg2	1.336993032
MYO4	IEY425	seg3	seg1	seg2	1.183518514
SNC1	IEY402	seg1	seg2	seg3	0.736815572
SNC1	IEY402	seg1	seg2	seg3	1.20226565
SNC1	IEY402	seg1	seg2	seg3	0.938507692
SNC1	IEY402	seg1	seg2	seg3	1.202826337
SNC1	IEY421	seg1	seg3	seg2	2.472528594
SNC1	IEY421	seg1	seg3	seg2	1.870532754
SNC1	IEY421	seg1	seg3	seg2	2.462705865
SNC1	IEY421	seg1	seg3	seg2	2.207198419
SNC1	IEY425	seg3	seg1	seg2	1.726046425
SNC1	IEY425	seg3	seg1	seg2	0.784421576
SNC1	IEY425	seg3	seg1	seg2	1.320609152
SNC1	IEY425	seg3	seg1	seg2	1.348257897

Supplementary Table 14: Gene expression ratio values of genes MYO4, SNC1 and NUP60 for ChrI restructured strains 1-3-2 (IEY421), 3-1-2 (IEY425) in relation to control strain 1-2-3 (IEY402). Column 1 ('Gene') lists IDs of each analyzed gene. Column 2 (‘Strain’) lists IDs of each analyzed strain. Column 3,4 and 5 ('Position 1','Position 2' and 'Position 3') lists the order of assembled segments for each restructured version of chromosome I. Column 5 ('Gene expression ratio') lists the Gene expression ratio of each analyzed gene per strain relative to the control strain IEY402. For each gene and strain 4 biological replicas were analyzed (n=4).

Gene	Strain 1	Strain 2	n1	n2	statistic	df	p	p.adj	p.adj.signif
MYO4	IEY402 (1-2-3)	IEY425 (3-1-2)	4	4	-3.6	6	0.011	0.034	*
MYO4	IEY402 (1-2-3)	IEY421 (1-3-2)	4	4	-3.3	6	0.017	0.035	*
MYO4	IEY425 (3-1-2)	IEY421 (1-3-2)	4	4	-2.0	6	0.093	0.093	ns
NUP60	IEY402 (1-2-3)	IEY425 (3-1-2)	4	4	1.4	6	0.202	0.202	ns
NUP60	IEY402 (1-2-3)	IEY421 (1-3-2)	4	4	-3.1	6	0.022	0.043	*
NUP60	IEY425 (3-1-2)	IEY421 (1-3-2)	4	4	-3.7	6	0.01	0.031	*
SNC1	IEY402 (1-2-3)	IEY425 (3-1-2)	4	4	-0.8	6	0.46	0.46	ns
SNC1	IEY402 (1-2-3)	IEY421 (1-3-2)	4	4	-6.8	6	0.000493	0.001	**
SNC1	IEY425 (3-1-2)	IEY421 (1-3-2)	4	4	-5.7	6	0.001	0.002	**

Supplementary Table 15: Two-tailed t-test results for *NUP60*, *MYO4* and *SNC1* expression ratios in restructured strains IEY402 (1-2-3), IEY421 (1-3-2), and IEY425 (3-1-2). The p-values were adjusted for multiple comparisons using Bonferroni correction (p.adj).

Cloning cassette	Sequence
Segment 1	GAATTCTTTCCGAATGACATGCGTCTCCTTGCGGGTAAATCACCGACCGCAA<br>TTCATAGAAGCCTGGGGGAACAGATAGGTCTAATTAGCTTAAGAGAGTAAATC<br>CTCATATGTACATTTATCGAGCCAATCGAGGGCAGCAGTTTAACATCAAGCCG<br>GATTTGCTCACGCTACTTTGACCCCTTTTCGTTTCGACGGAGAGAAGAAACC<br>GGTGTTTTCCTATCCTTGCCTCTCGAGGCGCGCATATCCTAGGTGGTTTCGA<br>TAGAGGCCAAGCGAATAATACTCCTTTTCCAGGTTATTTTGGAGGTTTGGATT<br>TATTTGATTATGACTTTTTGTTTGGCAATGACTTTGCTTAAAAATTTTCTTTCCA<br>AACTCCAGCCGCGCTATCCGGTAATCTCCAAATTAAAACATACCGTTCCATGA<br>AGGCTAGAATTACTTACCGGCCTTTTCCATGCCTGCGCTATACCCCCCCACT<br>CGCATGC
Segment 2	GAATTCAGCCGCGCTATCCGGTAATCTCCAAATTAAAACATACCGTTCCATGA<br>AGGCTAGAATTACTTACCGGCCTTTTCCATGCCTGCGCTATACCCCCCCACT<br>CAAATGCAACACTTCCCTAATATAGAAATTTGGGCATTAATTATTTTGAGAATTT<br>TGATGATTTGAATAATTTCATTAACGTAAAGGAACATAGTGCTACGAATCCAAC<br>AGTGGACCCAAAAATGACTCGAGGCGCGCATATCCTAGGATGCCTGCCACTA<br>GGAATCCATCGACGTACCATGGCTATAACTTTCCTTATGTTGTTTGCTTAGTTT<br>TTTGATATTAGTGTTGCTTATGTGAAATTTCGCGATTTCAATTAAAATAATAAATA<br>CATATGCATATGGTCCACAGGACACTCGTCGCTTTCGGATTTGCCCTCTATGT<br>GGCGGTTTTCAGGCACACTTATGCTCAGCACCGTTTAAACCAGACCGACGC<br>ATG
Segment 3	GAATTCTATGCATATGGTCCACAGGACACTCGTCGCTTTCGGATTTGCCCTCT<br>ATGTGGCGGTTTTCAGGCACACTTATGCTCAGCACCGTTTAAACCAGACCGA<br>CCTTTCCAGATTACAAGAACTTTGCGTTTGGCCTCTACAACGATTCTCACAAG<br>CATAAGGGCCATGCTGGTGTACAGGGAAATGTCTCTGCTGAGACACATTTCC<br>GGATTGAGATGGTCAGTAAAACTCGAGGCGCGCATATCCTAGGTTGGTGGTT<br>TAATCTATTTTTTATAAAAAATGACGCGGGCAGATTCAATTAGTGTCCTAAATTT<br>ATTCGCGTTTCAAGATTTCAAAGGATTGATCCTCTTATCAGAAACGATAAGTG<br>CTACTCCGAACTACGATGCCGCTAAGAACCTCTCGGTCGTCGCTGACGTTTA<br>CACTCTAGTCTCATTATAATCGTTCGCTATTCAGGGATTGACCAACACCGGAA<br>AACGCATG
Segment 4	GAATTCAACTACGATGCCGCTAAGAACCTCTCGGTCGTCGCTGACGTTTACA<br>CTCTAGTCTCATTATAATCGTTCGCTATTCAGGGATTGACCAACACCGGAAAA<br>CTTTTTTCATTTCTTATATTATTTTTTGTTCGAGAATCACTTTTTCAAGATGGTA<br>ACAACATCTTCGTCTTCCAAAATGTGACTCAACCCCACGTATTGAGGTTGATG<br>TTTGACACTGCTACCGTACTCGAGGCGCGCATATCCTAGGTGGTACAAGGAT<br>CTAAAAATGAAGATGTGTCTGGCTTTAGTAATCATCATATTGCTTGTTGTAATC<br>ATCGTCCCCATTGCTGTTCACTTTAGTCGATAGAAGTTCACTCGCAATGCTAT<br>ATATATGTCACTAGAGGACGCACGCTCTATTTTTATGATCCATTGATGTCCCTG<br>ACGCTGCAAAATTTGCAACCAGGCAGTCTTCGCGGTAGGTCCTAGTGCAAT<br>GGCATGC
Segment 5	GAATTCGTCACTAGAGGACGCACGCTCTATTTTTATGATCCATTGATGTCCCT<br>GACGCTGCAAAATTTGCAACCAGGCAGTCTTCGCGGTAGGTCCTAGTGCAA<br>TGAGGAATTGAGTATGCAGAATCAGAATAAAGGCTGACTTTCAAAAAAAGGTT<br>GTATTACAATTGCAGGTTTTCGATAAAAGAGACCCTATTCTCATCTACTACTGC<br>TAAACTTCGAGATATTTTCGCTCGAGGCGCGCATATCCTAGGCTGTTGCTGTT<br>GCTGCTGCGGGCCCACATTAGGGTAGCCTAGTAAAGAAGGGTCGTTCATTA<br>GCGGTAGTTTGCAGGGATAACGTCAGTCGGAGTTCCCTTGCTGGTGTCCCT

	TATGCTGTGCCCATACTCTTCCATAAACGGGCTATTAGTTATGAGGTCCGAAG<br>ATTGAAAAAGGTGAGGGAACTCGGCCGAACGGGAAAGACGGACATCTAGG<br>CAACCTGACGCATGC
Segment 6	GAATTCATACTCTTCCATAAACGGGCTATTAGTTATGAGGTCCGAAGATTGAA<br>AAAGGTGAGGGAACTCGGCCGAACGGGAAAGACGGACATCTAGGCAACCT<br>GACTGGCGTGTGCTATAGTGCTCTATATTCGAGTTTGTTGCTACTGGTGGAC<br>ACCCGACTATCTACAGTAAGGAACGTAAACAAGAAAAAGAGAGAAAATACGC<br>TATAGTTGAAAACATGAGTGGTTCCTCGAGGCGCGCATATCCTAGGTTGTTT<br>GATTTTGGTCCTTTTCTCCGGTAGGAGTTCTGATTCTGGCCCAGTTTCAGTC<br>TTTACCAGCGGTCTTTTCCTCAGAATTGCCATAGATGAGTATTTACTGATCTTT<br>TGCATATTTTTTTGTCGCTCTTGGGCCGCGGTGCGCTACCTTGCAGGAATTG<br>AGACCGTCCGTTAATTTCCCTTGCATATATATTGCGTTTCTTTGACCTTTTAAC<br>CGCTCTCGCATGC
Segment 7	GAATTGTCGCTCTTGGGCCGCGGTGCGCTACCTTGCAGGAATTGAGACCGT<br>CCGTTAATTTCCCTTGCATATATATTGCGTTTCTTTGACCTTTTAACCGCTCTC<br>CGGCAAAGTACAAGGGAAGGAAGCACAGAAGCAAGAGGAGGCGCATCGAT<br>CGTGGCAGATGAGTCAGCAAACACCACAGGAAAGTGAACAGACCACAGCG<br>AAAGAACAGGACCTTGATCAAGAGAGCTCGAGGCGCGCATATCCTAGGACT<br>CATAATATCTTGTCGAAAATACGCGGTGTAGGGAGTTATGGTGGATAACTTTT<br>TCACGATTAGAAGAAAAGGAAAATTTCATTATTCGTAGCTTAACATGGCAAAA<br>ACGAGAAAGACATATACAAAAGCTTAAATGGGAAATACGCGCCCATAACTTGG<br>TGCGAATACGGGTCGTAGCAATGTTCGTCTGACTATGATCTACATATTACAGG<br>CGGTACGTCTGCATGC
Segment 8	GAATTCCAAAAGCTTAAATGGGAAATACGCGCCCATAACTTGGTGCGAATAC<br>GGGTCGTAGCAATGTTCGTCTGACTATGATCTACATATTACAGGCGGTACGTC<br>TCATGAAGCGAAAGTACTTCACGACACCTAGATTGCAATCTACTCAATGTTAT<br>CCCTGGATGAAATATTATTTCGTTAACGACCATAGTAACTACCTGCTTCCATAT<br>GTTTGGCCTAATGGAACCACTCGAGGCGCGCATATCCTAGGAATCGTCAGTA<br>CCATTTTTCTTGTGGCTAGTTGGCTTCAACCAAACGTCCTCTTCTCTCTTATG<br>GCAAGAAGAAAGTTATATGTGTGACTGGTTGTTTATTTCACTTTCGCGACTGA<br>AAGCGCCGTCTAAACTAGAAAGTTACCGTTAGTCTTACTGATCCGAACCGTC<br>ATTAGTTACTAGAGGAAACGTGTCAAGAATGCCAGCCGATCCCACATTCTTAA<br>TTAGCATGC
Segment 9	GAATTCAATCAAAGGGAAGTCGAATAAAGGTCATCCAGGGAATTAGCCGTTT<br>TCGACCAAGTCTTTACGGGAGTAGAAGACGAATCCTCTCGTGTGCTGGCCC<br>AGGAAATTAAGATAGTTATTCGAGTGATTGCCAAATATCATGTTCTACTTCGAA<br>GACTTATAGCTAATTAATTTTTTCATAATGAAGGTGTCGTTAATTGTTCTGATTA<br>GTAACATGAAACTCAAAAACTCGAGGCGCGCATATCCTAGGAGTAGTTGTTTA<br>TCACTAGACATATAATTATGTTTATTTATATTTAGTGGGAGCAAAACAGTTTATT<br>GAATGTTTACCAGAACCGAAAAAAAAGCTCTTCTAAACTGTTGACATCCAGTT<br>CATTTTACTAGGTAACTAGAGGATTTGCGACGTTCTAAGCGTTGGTCCATGTG<br>AATCGCCATCCAGGATCACGTCGCCCTGAAAAAAAGATATCAGCAACTCTCC<br>GCATGC
Segment 10	GAATTCTACTAGGTAACTAGAGGATTTGCGACGTTCTAAGCGTTGGTCCATGT<br>GAATCGCCATCCAGGATCACGTCGCCCTGAAAAAAAGATATCAGCAACTCTC<br>CTACCCGTATGTATTAATGTATAAATGTTCTCAGAGCAAATTTTATCGATATCTT<br>GTTTGCCAGTGGTATGCAGGTTTGGCAAATTTTTTACCATAATATCCGTTTATA<br>GATTCTGGAACCTTACCCTCGAGGCGCGCATATCCTAGGCCTGAAAATTTGA<br>GGTTGTTCACGGAAATCATTTGGTTATGTCTGTCGGCCTGCTATTTAGAGACA<br>TTTTTTATTGCAACAACCTACTCTATGCACTTACACGGAATCGCAGAATAACG

	CGCCCATGCTCTAGGCATCTAACTATTCCCACTGCCTTAAGGGGGCCTGCGT<br>TTTCTGCCTGTCGATCCATAGGACTCGTGTCAACGCGCAGGCTTAGTTCGAG<br>ATGCATGC
Segment 11	GAATTCTGCTCTAGGCATCTAACTATTCCCACTGCCTTAAGGGGGCCTGCGT<br>TTTCTGCCTGTCGATCCATAGGACTCGTGTCAACGCGCAGGCTTAGTTCGAG<br>ATGGGTGTAAAAGTTATGTACGCTCGAAACAAATTTTATGTAGTTTACTTTAGA<br>TGCAAATGCTATTATATATTTTGCTTTATGATCCTCGGCTTGATGCTCGCCAAC<br>GTGAGATAGCTGGTCATCACTCGAGGCGCGCATATCCTAGGGACAATAACTG<br>GCAAGGGCTCTCACTAAATATCAACCCCTTTCAAATAAAAAAAGGATCATGGC<br>TGGCAGCGCCCACAACAATTAAACTCTGTTACTATCAAAAAACATTGAGCCCA<br>AGAATGGAACTTGATACCTAGTTAACCCACGGGTGTGGAAAAGCTCCCTAAG<br>GGAGGAACGGACGCCGGAAGGTTTTCCTGAGTGGCACTCACACATTATGGT<br>GTCAGGCATGC

Upstream adaptor; upstream homology arm; downstream homology; and downstream adaptor

Supplementary Table 16: Cloning cassettes for ChrI segment capture in *Saccharomyces cerevisiae* BY4742 used for multiplex deletion. Each module contains a pair of upstream and downstream adaptors flanking segment-specific homology arms that are separated by a site containing XhoI and AvrII sites used for vector linearization. Column 1 ('Cloning module') lists the ID of each cloned region. Column 2 ('Sequence') contains the nucleotide sequence of each specific clone module.

Segments	Start	End	Size (bp)
Deleted left sub-telomere	1649	31266	29617
Deleted segment 1	31267	34802	3535
Cloned segment 1	34803	51727	16925
Deleted segment 2	51728	52761	1033
Cloned segment 2	52762	56949	4188
Deleted segment 3	56950	57585	635
Cloned segment 3	57586	73851	16266
Deleted Segment 4	73852	75007	1155
Cloned segment 4	75008	87778	12771
Deleted segment 5	87779	92460	4681
Cloned segment 5	92461	113424	20964
Deleted segment 6	113425	114804	1379
Cloned segment 6	114805	128110	13306
Deleted segment 7	128111	129212	1101
Cloned segment 7	129213	143552	14340
Deleted segment 8	143553	147496	3943
Cloned segment 8	147497	151278	3781
Deleted Centromere	151279	151900	621
Cloned segment 9	151901	160045	8144
Deleted segment 9	160046	170356	10310
Cloned segment 10	170357	182386	12029
Deleted segment 10	182387	184582	2195
Cloned segment 11	184583	196952	12369
Deleted right sub-telomere	197029	229121	32092

Supplementary Table 17: Location and size of each target segment on ChrI for cloning or deletion during multiplex deletion assay. Column 1 ('Segment') lists the IDs of all target segments. Column 2 ('Start') lists the start position of each segment referent to *S.cerevisiae* ChrI coordinates. Column 3 ('End') lists the end position for each segment. Column 4 ('Size(bp)') lists the size of each segment.

Feature	Feature Systematic Name	Feature Type	Coordinates	Location
GDH3	*YAL062W*	ORF	31567-32940	Deleted segment 1
BDH2	*YAL061W*	ORF	33448-34701	Deleted segment 1
AIM2	*YAL049C*	ORF	51855-52595	Deleted segment 2
BOL3	*YAL046C*	ORF	57029-57385	Deleted segment 3
YAL037W	*YAL037W*	ORF	74020-74823	Deleted segment 4
MYO4	*YAL029C*	ORF	87855-92270	Deleted segment 5
ATS1	*YAL020C*	ORF	113614-114615	Deleted segment 6
SYN8	*YAL014C*	ORF	128252-129019	Deleted segment 7
VPS8	*YAL002W*	ORF	143707-147531	Deleted segment 8
YARCdelta3	*YARCdelta3*	Long terminal repeat	160105-160237	Deleted segment 9
YARCTy1-1	*YARCTy1-1*	LTR retrotransposon	160238-166162	Deleted segment 9

TGA1	*YNCA0004W*	tRNA gene	166267-166339	Deleted segment 9
BUD14	*YAR014C*	ORF	166742-168871	Deleted segment 9
ADE1	*YAR015W*	ORF	169375-170295	Deleted segment 9
YNCA0006C	*YNCA0006C*	tRNA gene	182522-182603	Deleted segment 10
YARWsigma1	*YARWsigma1*	Long terminal repeat	182620-182959	Deleted segment 10
YARWdelta6	*YARWdelta6*	Long terminal repeat	183142-183474	Deleted segment 10
UIP3	*YAR027W*	ORF	183770-184477	Deleted segment 10

Supplementary Table 18: Genetic elements removed from the ChrI multiple deletion strain. Column 1 ('Features') lists the standard ID of each genetic element removed from ChrI. Column 2 ('Systematic feature') lists the systematic feature of each genetic element removed from ChrI. Column 3 ('Feature type') associates the type of feature to each genetic element. Column 4 ('Coordinates') lists the start and end position of each removed element based on ChrI sequence coordinates. Column 5 ('Location') associates each removed genetic feature to a deleted segment into multiple deleted ChrI strain. A given deleted segment may contain one or more listed features.

gRNAs	Sequence
Segment 1 down	AATTGATGAAATGAATAGGT
Segment 1 down	ATATATATATATGTATATAA
Segment 1 up	ACGTTCTCGTGTTAATCCCG
Segment 1 up	TTAGTAAAACAAGAAGACCG
Segment 10 down	CAACACAATTGGGAAACGAT
Segment 10 down	AAGCATACTCATCTTCAACT
Segment 10 up	TATTCAGTGAGGAGTTACAC
Segment 10 up	TGTATGAATCATATTCAGTG
Segment 11 up	GATGGACTGACACTTAATTG
Segment 11 up	AATTAAGTGTCAGTCCATCT
Segment 11 down	ATAAAATTTTCACTACACCT
Segment 11 down	CATGTACAAATCCATGTCCG
Segment 2 down	TATATAAAGAATATACACAG
Segment 2 down	ATATAAAGAATATACACAGA
Segment 2 up	TCTGCTGTAGTTAGACGTAG
Segment 2 up	CTATGATATCTGGCCTAAGG
Segment 3 down	AGATGGCTAGAAGAATAGGA
Segment 3 down	AGATGGGAAGAAAACTGAAG
Segment 3 up	ATCCTGAAGAGACTGGAATG
Segment 3 up	GCATTCCAGTCTCTTCAGGA
Segment 4 down	GGAGCTTCCATATGTAGTGT
Segment 4 down	CATATATCCTACACTACATA
Segment 4 up	AGCATTTGTATTCGAAAAGG
Segment 4 up	AAGCATTTGTATTCGAAAAG

Segment 5 down	GAGAAGGTTCTCAAGCACAA
Segment 5 down	CGATGAGAATTAATGCTGAA
Segment 5 up	AAAAAAGGTGATAACTCCGT
Segment 5 up	TGATAACTCCGTAGGAATTG
Segment 6 down	TATATGATGAATCATTAAAG
Segment 6 down	ATGATGAATCATTAAAGAGG
Segment 6 up	CAGATCCAAAGCGCAAGTGA
Segment 6 up	CACGTAACTTAAATCAGTAC
Segment 7 down	ATCAAAACGTGAGTTTCCTG
Segment 7 down	AGGTATATCATCGTAACCAG
Segment 7 up	CACGACTTTGATCACCCGAA
Segment 7 up	ATAAACTCATAACAGTGCAA
Segment 8a down	TTATGATGCAAAAAACCAAG
Segment 8a down	CTGCATTTTCAAATACCGCT
Segment 8a up	TGAATCTACATAATGAAATG
Segment 8a up	AAATATCAGAAATATGCAAA
Segment 9 down	ACTTCCACGTGTAGATGTGA
Segment 9 down	TGTTCCTTCACATCTACACG
Segment 9 up	CACCTTTCGTCTGCTGCCTC
Segment 9 up	ATAATTTGAAAAAACACCCG

Supplementary Table 19: gRNAs used to clone segments for the multiplex deletion experiment. Column 1 ('gRNAs') lists the gRNAs IDs. The ‘up’ and ‘down’ annotations refer to the side of a segment targeted by a guide, Column 2 ('sequence') lists the target sequence of each gRNA.

Primers	Sequence
GDH3 check REV	ggaagcgtaggccaccct
Seg1 capture confirm REV	gagccacaaatcccacacca
Seg1 capture confirm FOR	agagcttggactacaacagtgg
AIM2 check FOR	gagatgtagagcctgctgca
Seg2 capture confirm REV	acccactgcacatatcatccag
Seg2 capture confirm FOR	atcagtgtcattgcccagcg
BOL3 check REV	tctcatcaagcagcaccagc
Seg3 capture confirm REV	tggtgagggcgttaatgtgg
Seg3 capture confirm FOR	ggactgatgatgttgaagccc
YAL037W check REV	tgctgctgtagttggtgagc
Seg4 capture confirm REV	tgccgtgcctatttcgtctgg
Seg4 capture confirm FOR	ggactgcggaactacaagctg
MYO4 check REV	aagcctgctaacaagggcga
Seg5 capture confirm REV	gctttacatcttccaggggtct
Seg5 capture confirm FOR	gagcatgttcgcgttcccat
ATS1 check REV	cgcgcctcaatacggtagag
Seg6 capture confirm REV	ggggaactctctcgcaaggt
Seg6 capture confirm FOR	cgacgggggctagaatcttaga
SYN8 check REV	gtcacagtcacagccacagc

Seg7 capture confirm REV	gctggatagcttgggagcg
Seg7 capture confirm FOR	gccagctgctaagtccattgtc
VPS8 check REV	gtcggcactaggattgtctgc
Seg8 capture confirm REV	caaagttccactgggcaaacc
Seg8 capture confirm FOR	tcccgttaataatgtccataggct
Seg9 capture confirm REV	atagggagcggaaggcacag
Seg9 capture confirm FOR	agcagtacagacaggcagga
BUD14 check FOR	agtctcatcaacatgctcttcct
Seg10 capture confirm REV	acccaggcagtggaggag
Seg10 capture confirm FOR	cgctcgccatgagatatgct
UIP3 check FOR	tacgcagtcaaacacaccagc
Seg11 capture confirm REV	ctgaaggtgtttgcatggtca
Seg11 capture confirm FOR	ccatggattcaaagccgatacc

Supplementary Table 20: Primers used to confirm capture of segments 1 through 11 on multiple deletion assay. Column 1 (‘Primers’) lists primers IDs. Column 2 (‘Sequence’) contains the nucleotide sequence of each primer.

Feature	Feature Systematic Name	Feature Type	Coordinates	Location
YAL069W	*YAL069W*	ORF	335-649	Left Sub telomere
YAL068W-A	*YAL068W-A*	ORF	538-792	Left Sub telomere
PAU8	*YAL068C*	ORF	1807-2169	Left Sub telomere
YAL067W-A	*YAL067W-A*	ORF	2480-2707	Left Sub telomere
SEO1	*YAL067C*	ORF	7235-9016	Left Sub telomere
YAL066W	*YAL066W*	ORF	10091-10399	Left Sub telomere
YAL065C	*YAL065C*	ORF	11565-11951	Left Sub telomere
YAL064W-B	*YAL064W-B*	ORF	12046-12426	Left Sub telomere
TDA8	*YAL064C-A*	ORF	13363-13743	Left Sub telomere
YAL064W	*YAL064W*	ORF	21566-21850	Left Sub telomere
YALWdelta1	*YALWdelta1*	long terminal repeat	22230-22552	Left Sub telomere
YAL063C-A	*YAL063C-A*	ORF	22395-22685	Left Sub telomere
FLO9	*YAL063C*	ORF	24000-27968	Left Sub telomere
YAR047C	*YAR047C*	ORF	201467-201787	Right Sub telomere

FLO1	*YAR050W*	ORF	203403-208016	Right Sub telomere
YAR053W	*YAR053W*	ORF	208367-208663	Right Sub telomere
YARCdelta8	*YARCdelta8*	long terminal repeat	209448-209778	Right Sub telomere
YAR060C	*YAR060C*	ORF	217157-217492	Right Sub telomere
YAR061W	*YAR061W*	pseudogene	218140-219145	Right Sub telomere
YAR064W	*YAR064W*	ORF	220198-220497	Right Sub telomere
YAR066W	*YAR066W*	ORF	221049-221660	Right Sub telomere
YAR068W	*YAR068W*	ORF	222406-222891	Right Sub telomere
YAR069C	*YAR069C*	ORF	224011-224304	Right Sub telomere
YAR070C	*YAR070C*	ORF	224563-224862	Right Sub telomere
PHO11	*YAR071W*	ORF	225460-226863	Right Sub telomere
IMD1	*YAR073W*	ORF	227742-228953	Right Sub telomere
YAR075W	*YAR075W*	ORF	228844-229317	Right Sub telomere

Supplementary Table 21: Genetic elements present on both sub-telomeric regions in *S.cerevisiae* BY ChrI. Both sub-telomeres (left and right) and consequently their genetic elements were removed from all our synthetic ChrI constructs. Column 1 ('Features') lists the standard ID of each genetic element present on sub-telomeres. Column 2 ('Systematic feature') lists the systematic feature of each genetic element present on sub-telomeres. Column 3 ('Feature type') associates the type of feature to each genetic element. Column 4 ('Coordinates') lists the start and end position of each element based on ChrI

sequence coordinates. Column 5 ('Location') lists if a given feature is located on the left or right sub-telomere on ChrI.

Strain	Doubling Time at 30ºC (min)
BY4742	88.72931969
BY4742	83.39266681
BY4742	82.89834573
BY4742	83.50118352
BY4742	89.13436854
BY4742	91.37895703
BY4742	91.16826909
BY4742	91.6830047
BY *syn8Δ*	87.98780256
BY *syn8Δ*	81.92212109
BY *syn8Δ*	87.85825647
BY *syn8Δ*	85.44066121
BY *syn8Δ*	92.91753156
BY *syn8Δ*	90.06264858
BY *syn8Δ*	88.07455688
BY *syn8Δ*	86.94347991
BY *syn8Δ*	87.77750448
BY synthetic ChrI	88.24396232
BY synthetic ChrI	93.65803672
BY synthetic ChrI	95.50624222
BY synthetic ChrI	97.04904224
BY synthetic ChrI	94.27469593
BY synthetic ChrI	89.61415658

BY synthetic ChrI	86.72118957
BY synthetic ChrI	94.01112848
BY synthetic ChrI	95.84090862
ChrI multiple deletion	219.094345
ChrI multiple deletion	214.1159012
ChrI multiple deletion	225.582891
ChrI multiple deletion	208.6559921
ChrI multiple deletion	200.8680417
ChrI multiple deletion	225.2619949
ChrI multiple deletion	210.2813049
ChrI multiple deletion	208.9293032
ChrI multiple deletion	235.2457741
ChrI multiple deletion	244.3415846
ChrI multiple deletion	277.1097999
ChrI multiple deletion	230.0858969
ChrI multiple deletion *syn8*Δ	437.1075259
ChrI multiple deletion *syn8*Δ	424.9516265
ChrI multiple deletion *syn8*Δ	396.5195141
ChrI multiple deletion *syn8*Δ	399.462152
ChrI multiple deletion *syn8*Δ	390.0608333
ChrI multiple deletion *syn8*Δ	386.8709313
ChrI multiple deletion *syn8*Δ	389.6391713
ChrI multiple deletion *syn8*Δ	390.8815326

Supplementary Table 22: Phenotypic assay of strains generated during ChrI multiple deletion assay and their controls. Column I ('Strain') lists IDs of each strain. The 'ChrI multiple deletion strain' contains a version of ChrI where 15 out of the 16 targeted genetic elements were removed in a single yeast

transformation. The 'ChrI multiple deletion strain *syn8Δ*' is the former 'ChrI multiple deletion strain' whose gene *SYN8* was deleted through CRISPR/cas9 cutting and replacement. The 'BY synthetic ChrI strain' contains a circular assembled version of ChrI without the sub-telomeres but preserved core region. The 'BY *syn8Δ*' strain is the parental wild-type BY4742 strain whose gene *SYN8* was deleted by using CRISPR/CAS9 cutting and replacement. The 'BY4742 strain' is the wild type parental *S.cerevisiae* strain. Column 2 ('Doubling time at 30ºC (min)') lists the doubling time of each strain growing on rich media at 30ºC.

gRNA	Sequence
SYN8 gRNA1	ggacaaaattcctgatacgg
SYN8 gRNA2	ggatgacgatgaacaagacg
pASC2 gRNA up 1	gcgtagggaacgtcgcaaag
pASC2 gRNA up 2	ccgcacagatgcgtaaggta
pASC2 gRNA down 1	ggtgtggaaaagctccctaa
pASC2 gRNA down 2	tatccctactcactcaaagg

Supplementary Table 23: gRNAs used during *S.par* ChrI linearization and *SYN8* deletion. Column 1 ('gRNAs') lists the gRNAs IDs. The 'up' and 'down' annotations refer to the side of a segment targeted by a guide, Column 2 ('sequence') lists the target sequence of each gRNA.

Uncropped gel images used in Supplementary Figures

Supplementary Figure 7

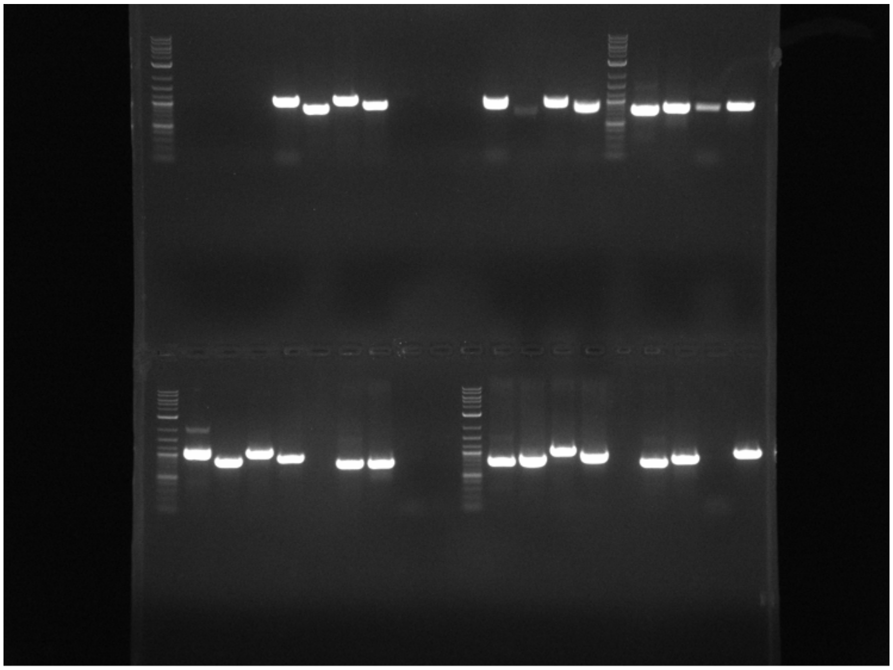

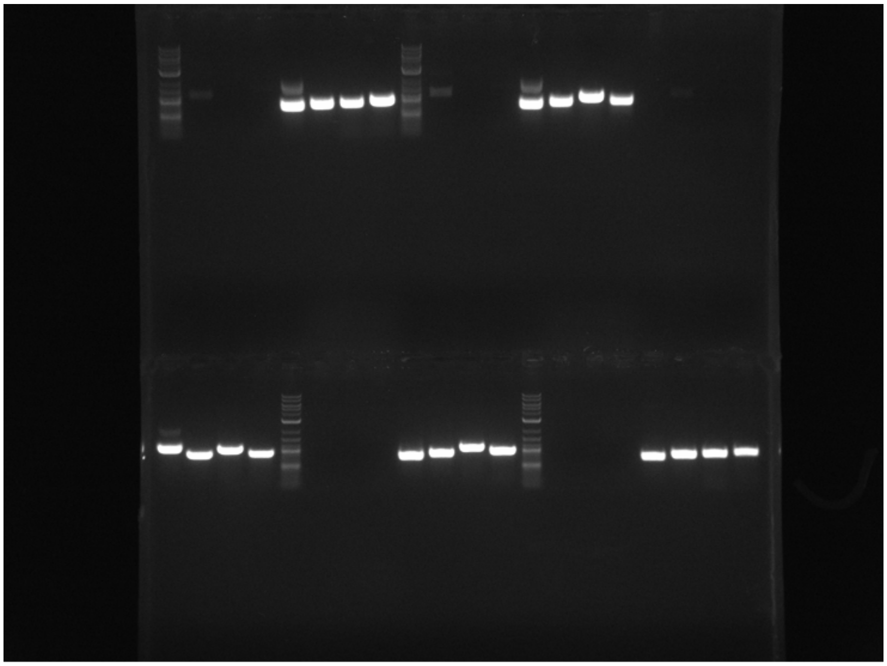

Supplementary Figure 11

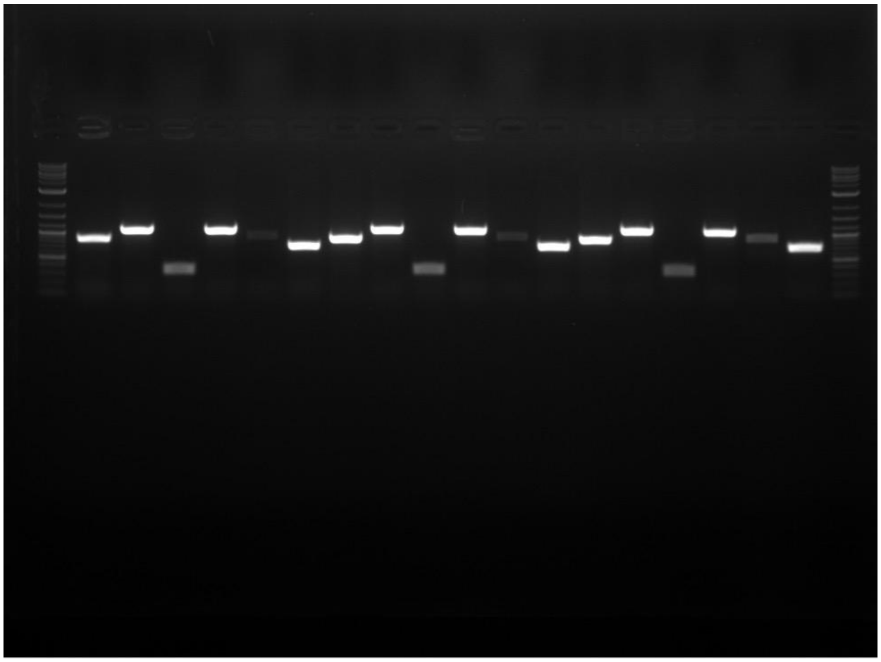

Supplementary Figure 12

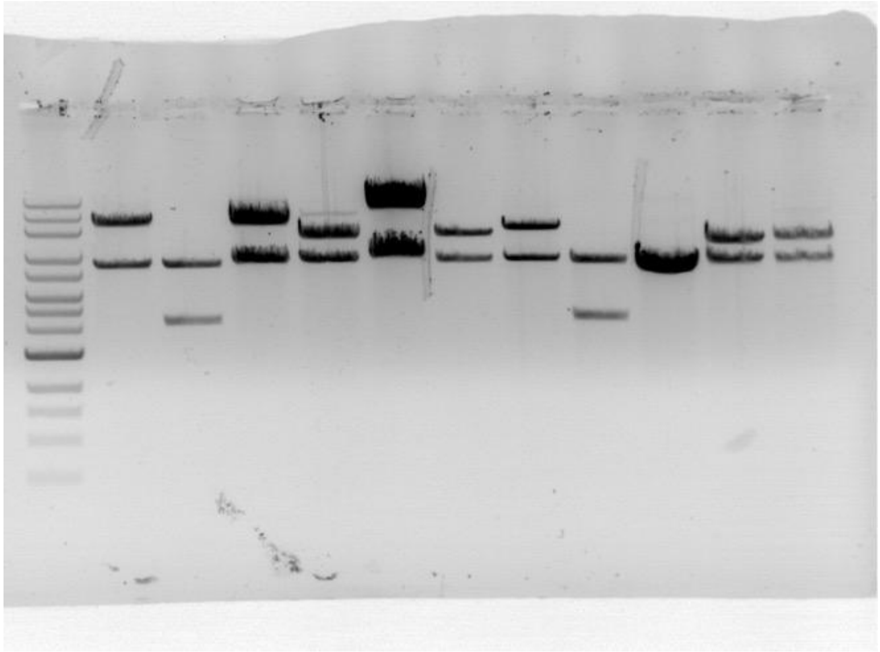

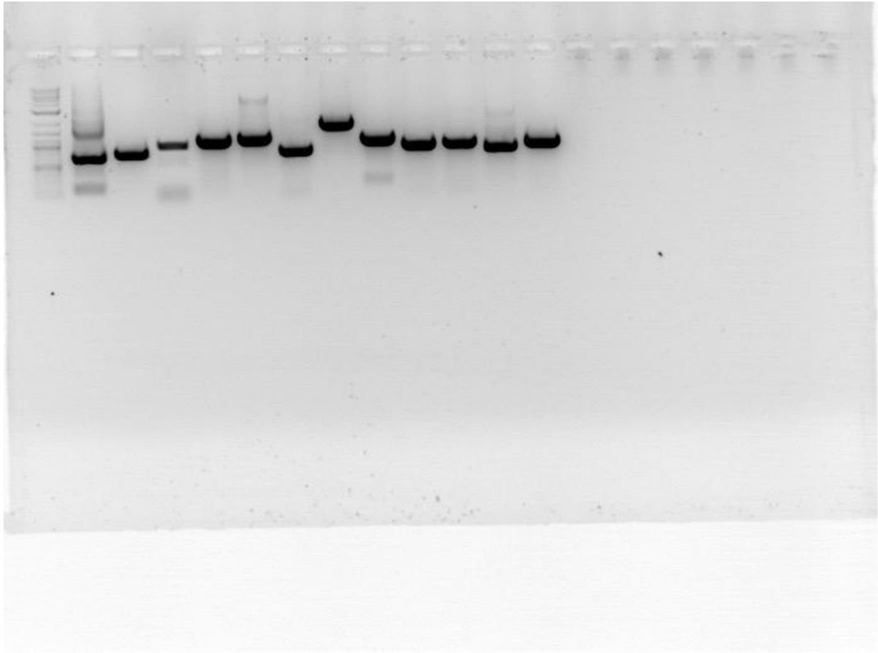

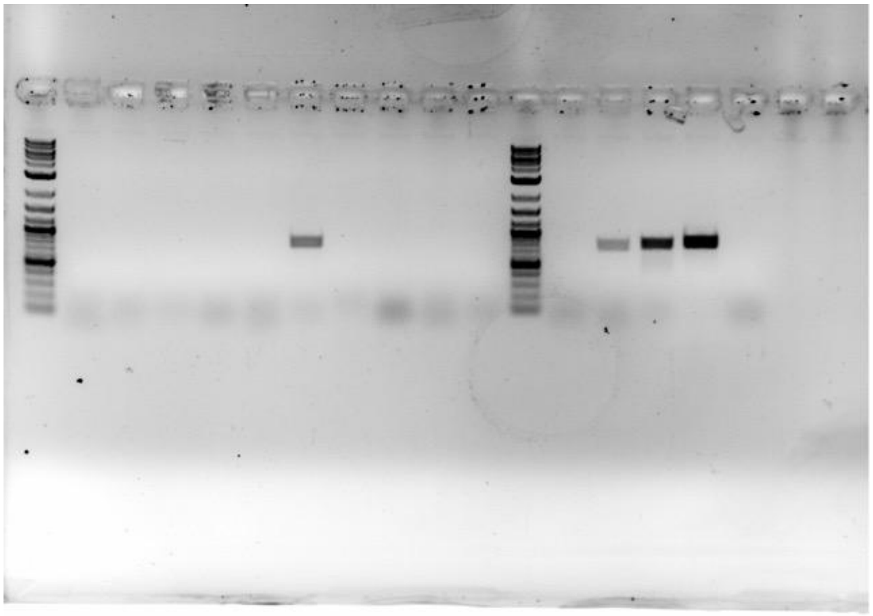

Supplementary Figure 13

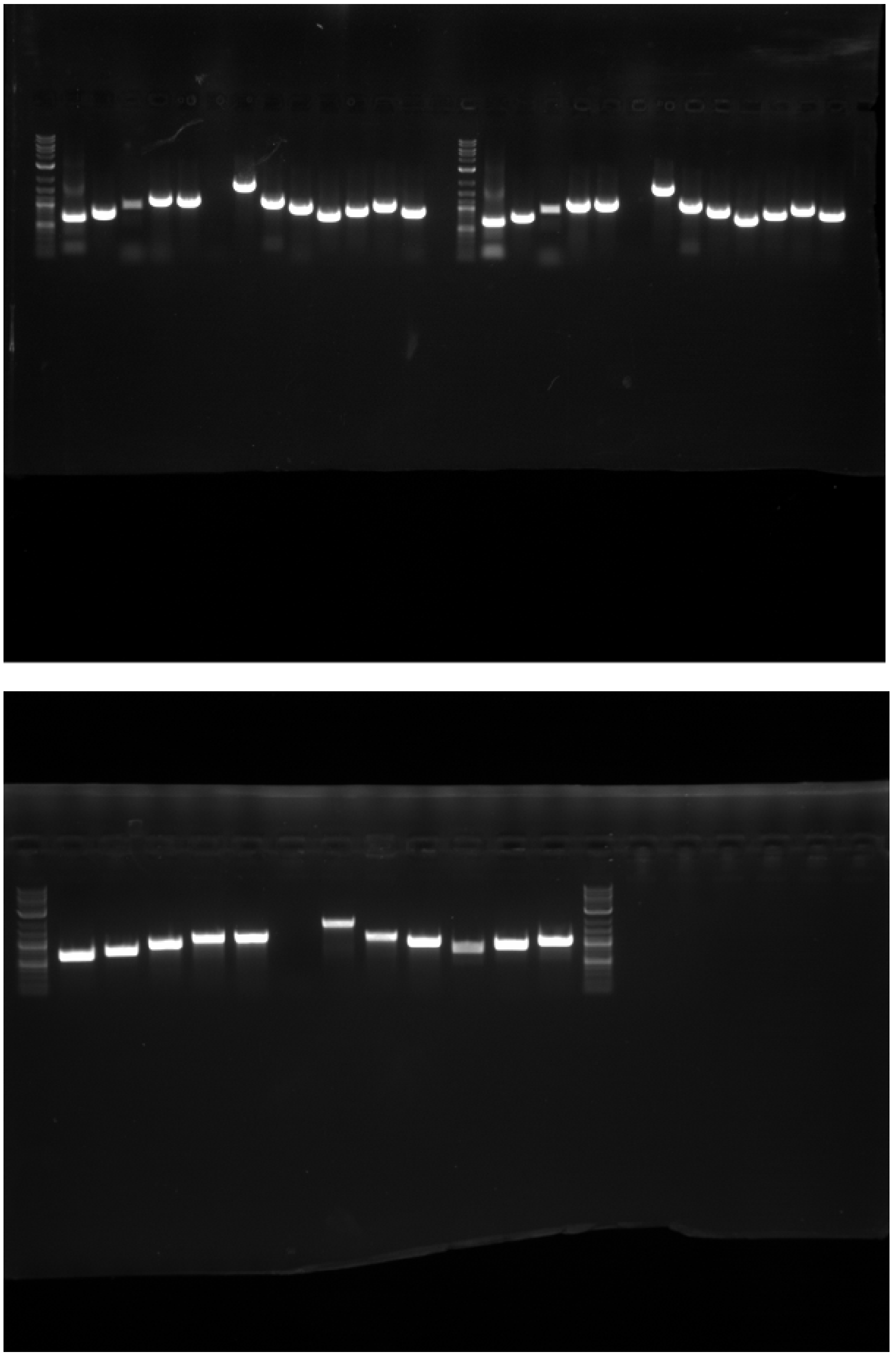

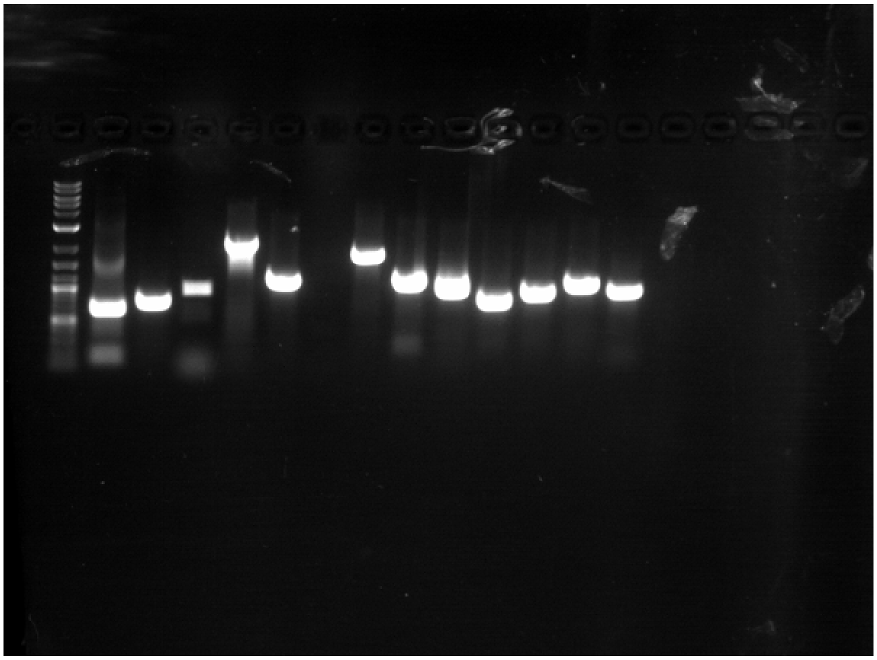

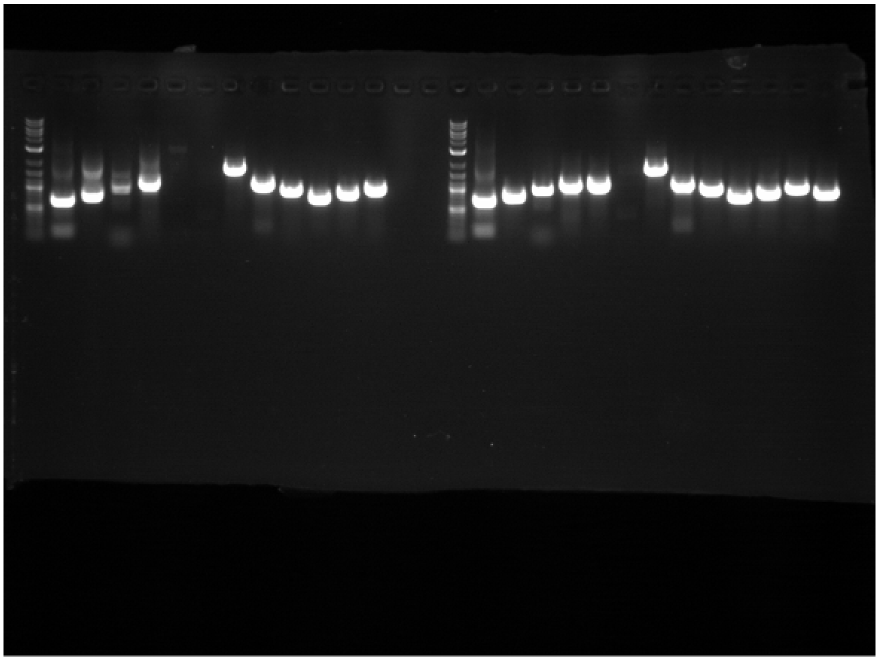

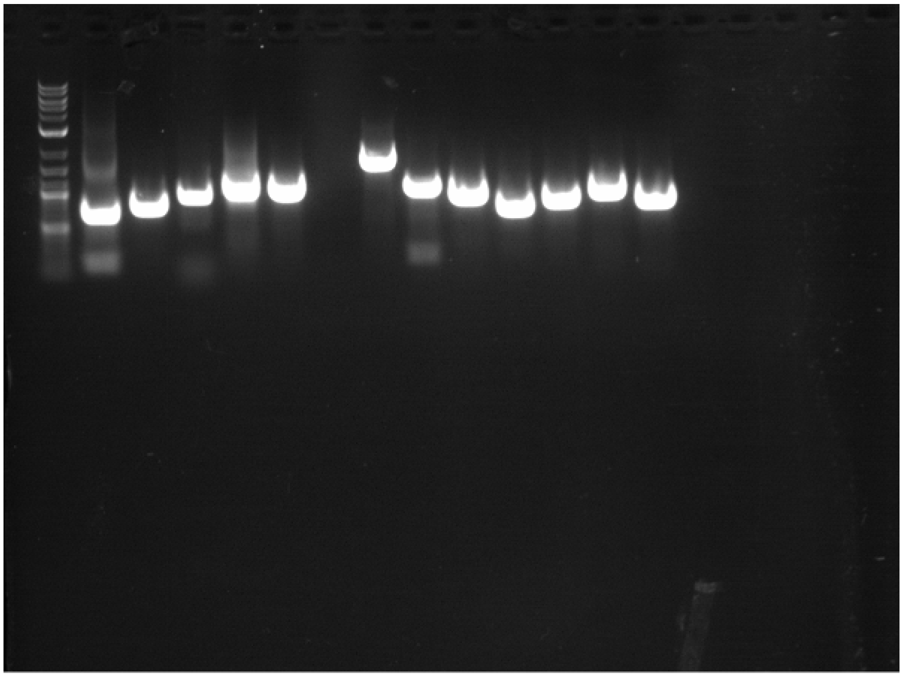

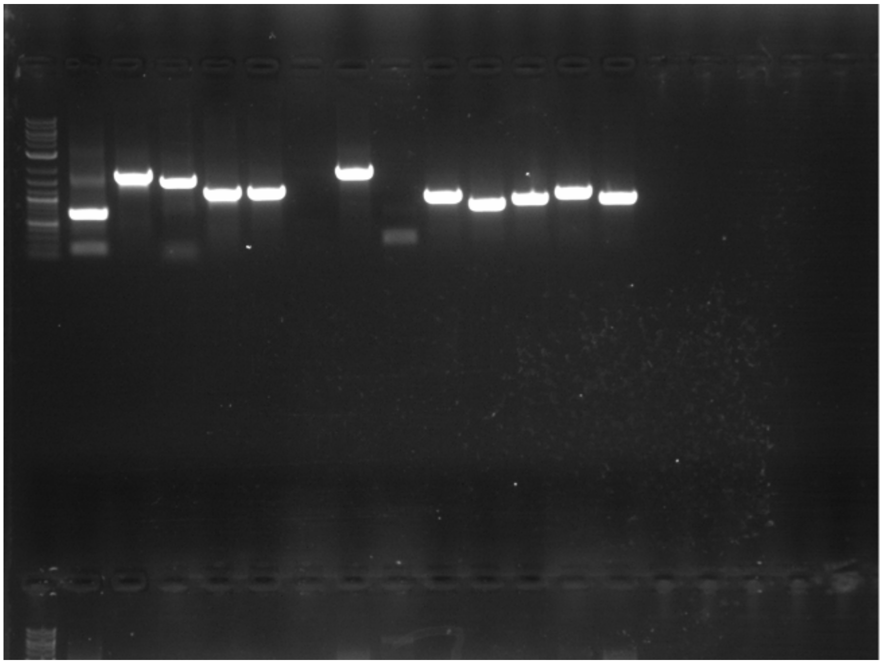
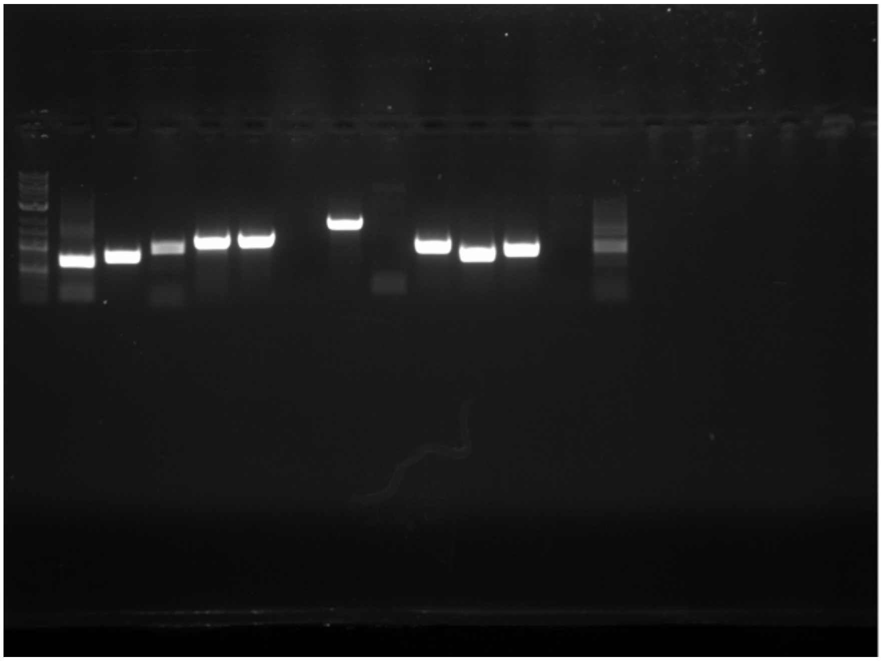

Supplementary Figure 14

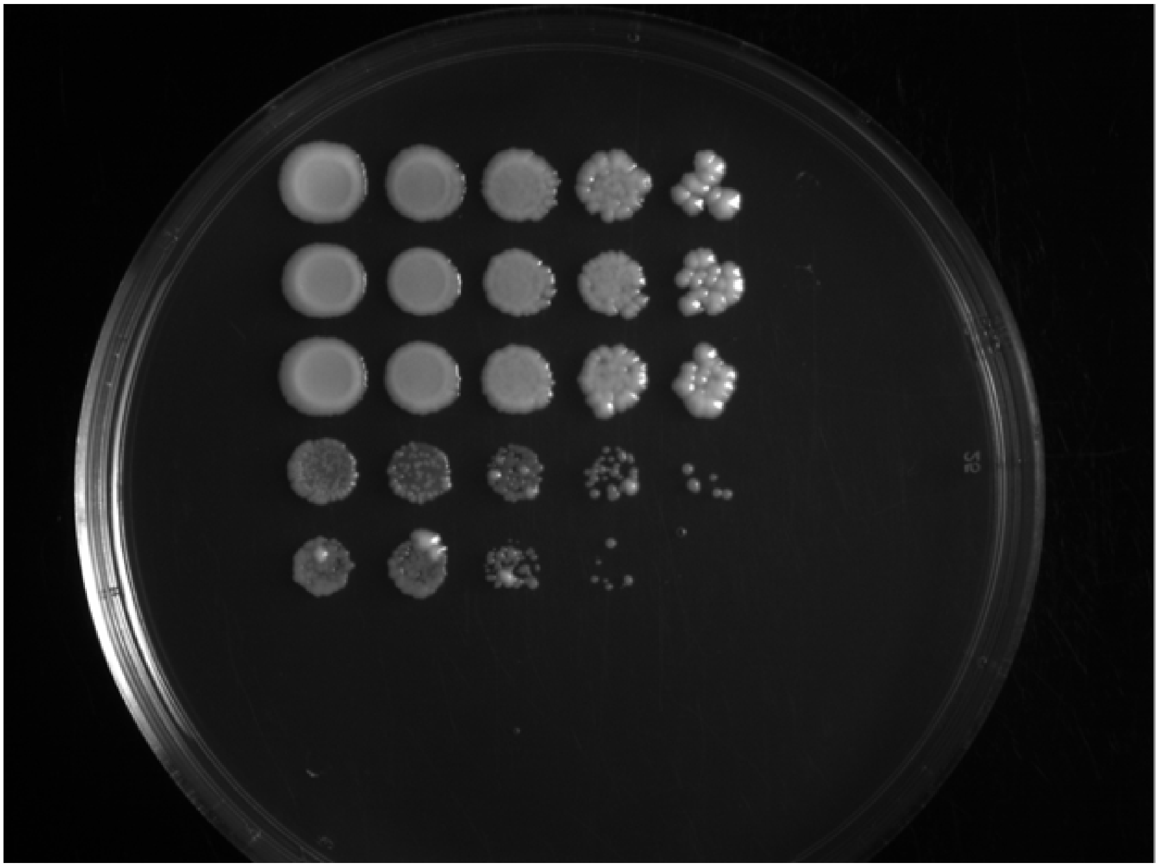

Supplementary Figure 15

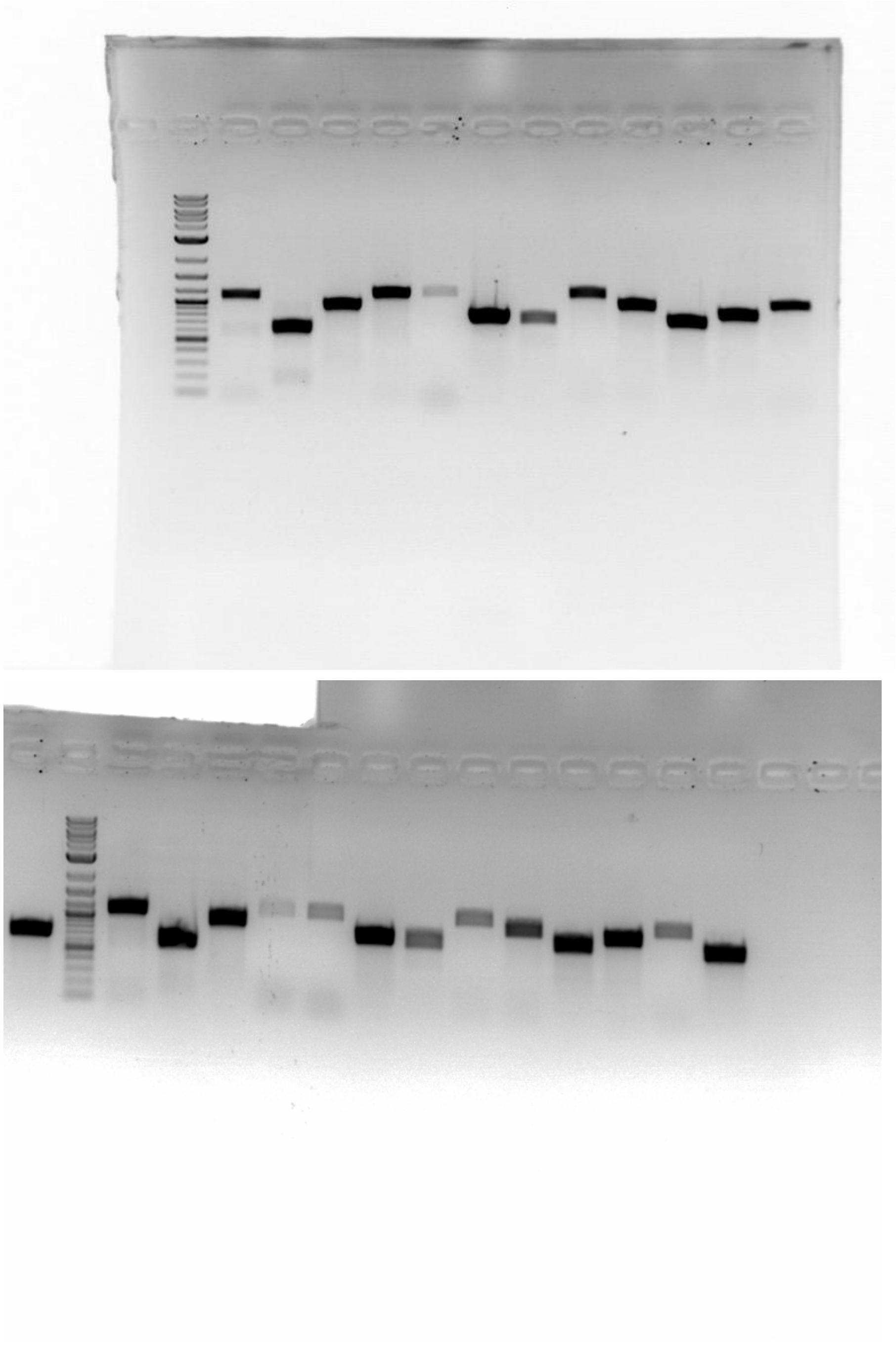

Supplement: Supplementary file 1 — Supplementary Information [file 41467_2023_44112_MOESM1_ESM.pdf]
