# Supplementary material for: Building synthetic chromosomes from natural DNA
Source: Nat Commun. 2023 Dec 20;14:8337. doi: 10.1038/s41467-023-44112-2 (PMC10733283; doi:10.1038/s41467-023-44112-2)
Supplement: Supplementary file 3 — Reporting Summary [file 41467_2023_44112_MOESM3_ESM.pdf]

nature portfolio

Corresponding author(s): Ehrenreich, IM
Coradini, ALV

Last updated by author(s): November 26, 2023

Reporting Summary

Nature Portfolio wishes to improve the reproducibility of the work that we publish. This form provides structure for consistency and transparency in reporting. For further information on Nature Portfolio policies, see our Editorial Policies and the Editorial Policy Checklist.

Statistics

For all statistical analyses, confirm that the following items are present in the figure legend, table legend, main text, or Methods section.

n/a	Confirmed	
☐	☒	The exact sample size (*n*) for each experimental group/condition, given as a discrete number and unit of measurement
☐	☒	A statement on whether measurements were taken from distinct samples or whether the same sample was measured repeatedly
☐	☒	The statistical test(s) used AND whether they are one- or two-sided<br>*Only common tests should be described solely by name; describe more complex techniques in the Methods section.*
☐	☒	A description of all covariates tested
☐	☒	A description of any assumptions or corrections, such as tests of normality and adjustment for multiple comparisons
☐	☒	A full description of the statistical parameters including central tendency (e.g. means) or other basic estimates (e.g. regression coefficient) AND variation (e.g. standard deviation) or associated estimates of uncertainty (e.g. confidence intervals)
☐	☒	For null hypothesis testing, the test statistic (e.g. *F*, *t*, *r*) with confidence intervals, effect sizes, degrees of freedom and *P* value noted<br>*Give P values as exact values whenever suitable.*
☒	☐	For Bayesian analysis, information on the choice of priors and Markov chain Monte Carlo settings
☐	☒	For hierarchical and complex designs, identification of the appropriate level for tests and full reporting of outcomes
☐	☒	Estimates of effect sizes (e.g. Cohen's *d*, Pearson's *r*), indicating how they were calculated

Our web collection on statistics for biologists contains articles on many of the points above.

Software and code

Policy information about availability of computer code

Data collection	No software were used for data collection.
Data analysis	All software were open source. Sequencing data was analyzed using the following programs: minimap2 (v. 2.24-r1122, -ax map-ont); samtools (v1.15, htslib v1.15, view -bS); bamtools (v2.5.2); Nanocaller (v3); and Sniffles (v2.2). Data analysis was performed using R (v4.0.2); Python (v3.7.6); PRECOG (ver 1.0.0.16); and AriaMx qPCR Software (v2.1).

For manuscripts utilizing custom algorithms or software that are central to the research but not yet described in published literature, software must be made available to editors and reviewers. We strongly encourage code deposition in a community repository (e.g. GitHub). See the Nature Portfolio guidelines for submitting code & software for further information.

Data

Policy information about availability of data

All manuscripts must include a data availability statement. This statement should provide the following information, where applicable:

- Accession codes, unique identifiers, or web links for publicly available datasets
- A description of any restrictions on data availability
- For clinical datasets or third party data, please ensure that the statement adheres to our policy

All data generated or analyzed in this study are included with this manuscript via Figshare (https://doi.org/10.6084/m9.figshare.24251383). Raw sequencing data is available at the NCBI Sequence Read Archive under the accession PRJNA986901.

Research involving human participants, their data, or biological material

Policy information about studies with human participants or human data. See also policy information about sex, gender (identity/presentation), and sexual orientation and race, ethnicity and racism.

Reporting on sex and gender	N/A
Reporting on race, ethnicity, or other socially relevant groupings	N/A
Population characteristics	N/A
Recruitment	N/A
Ethics oversight	N/A

Note that full information on the approval of the study protocol must also be provided in the manuscript.

Field-specific reporting

Please select the one below that is the best fit for your research. If you are not sure, read the appropriate sections before making your selection.

☒ Life sciences ☐ Behavioural & social sciences ☐ Ecological, evolutionary & environmental sciences

For a reference copy of the document with all sections, see nature.com/documents/nr-reporting-summary-flat.pdf

Life sciences study design

All studies must disclose on these points even when the disclosure is negative.

Sample size	We included all generated strains in all relevant phenotyping experiments. Thus, the sample size was always all strains.
Data exclusions	All generated data was included in analyses.
Replication	All yeast growth measurements are based on at least nine biological replicates.
Randomization	Completely randomized ordering and/or positioning was used during phenotyping experiments. For example, in a 96-well plate, the rows and columns occupied by each strain were determined by assigning a number to each strain and using a random number generator to order the assigned numbers.
Blinding	Blinding was not necessary in this study as there were no subjective outcomes possible in our experiments. All strains were phenotyped in all conditions, all inferences were made based on randomized experiments, and all statistical tests were performed without a priori hypotheses using rigorous thresholds.

Reporting for specific materials, systems and methods

We require information from authors about some types of materials, experimental systems and methods used in many studies. Here, indicate whether each material, system or method listed is relevant to your study. If you are not sure if a list item applies to your research, read the appropriate section before selecting a response.

Materials & experimental systems

n/a	Involved in the study	
☒	☐	Antibodies
☐	☒	Eukaryotic cell lines
☒	☐	Palaeontology and archaeology
☒	☐	Animals and other organisms
☒	☐	Clinical data
☒	☐	Dual use research of concern
☒	☐	Plants

Methods

n/a	Involved in the study	
☒	☐	ChIP-seq
☒	☐	Flow cytometry
☒	☐	MRI-based neuroimaging

Eukaryotic cell lines

Policy information about cell lines and Sex and Gender in Research

Cell line source(s)	The Saccharomyces cerevisiae BY4742 strain was obtained from the American Type Culture Collection. The Saccharomyces cerevisiae RM11-1a strain and the Saccharomyces paradoxus CBS5829 strain were obtained from the authors of Liti et al. 2009. Population genomics of domestic and wild yeasts. All three strains are commonly used in the budding yeast research community and are available from many labs, including the Ehrenreich lab.
Authentication	Strains were authenticated by Oxford Nanopore Technologies whole genome sequencing and comparison of sequencing data against previously published genomes.
Mycoplasma contamination	The cell lines were not tested for Mycoplasma contamination, as such contamination is not an issue for yeast cultures.
Commonly misidentified lines (See ICLAC register)	N/A

Plants

Seed stocks	N/A
Novel plant genotypes	N/A
Authentication	N/A
